# Genetic diversity and selection in Puerto Rican horses

**DOI:** 10.1038/s41598-021-04537-5

**Published:** 2022-01-11

**Authors:** Walter W. Wolfsberger, Nikole M. Ayala, Stephanie O. Castro-Marquez, Valerie M. Irizarry-Negron, Antoliy Potapchuk, Khrystyna Shchubelka, Ludvig Potish, Audrey J. Majeske, Luis Figueroa Oliver, Alondra Diaz Lameiro, Juan Carlos Martínez-Cruzado, Gabriella Lindgren, Taras K. Oleksyk

**Affiliations:** 1 Department of Biological Sciences, Oakland University, Rochester, MI USA; 2 Biology Department, University of Puerto Rico at Mayaguez, Mayaguez, Puerto Rico; 3 Biology Department, Uzhhorod National University, Uzhhorod, Ukraine; 4 Department of Forestry, Uzhhorod National University, Uzhhorod, Ukraine; 5 Department of Animal Breeding and Genetics, Swedish University of Agricultural Sciences, Uppsala, Sweden; 6 Livestock Genetics, Department of Biosystems, KU Leuven, Leuven, Belgium

**Keywords:** Animal breeding, Evolutionary genetics, Agricultural genetics, Population genetics, Evolution

## Abstract

Since the first Spanish settlers brought horses to America centuries ago, several local varieties and breeds have been established in the New World. These were generally a consequence of the admixture of the different breeds arriving from Europe. In some instances, local horses have been selectively bred for specific traits, such as appearance, endurance, strength, and gait. We looked at the genetics of two breeds, the Puerto Rican Non-Purebred (PRNPB) (also known as the “Criollo”) horses and the Puerto Rican Paso Fino (PRPF), from the Caribbean Island of Puerto Rico. While it is reasonable to assume that there was a historic connection between the two, the genetic link between them has never been established. In our study, we started by looking at the genetic ancestry and diversity of current Puerto Rican horse populations using a 668 bp fragment of the mitochondrial DNA D-loop (HVR1) in 200 horses from 27 locations on the island. We then genotyped all 200 horses in our sample for the “gait-keeper” *DMRT3* mutant allele previously associated with the paso gait especially cherished in this island breed. We also genotyped a subset of 24 samples with the Illumina Neogen Equine Community genome-wide array (65,000 SNPs). This data was further combined with the publicly available PRPF genomes from other studies. Our analysis show an undeniable genetic connection between the two varieties in Puerto Rico, consistent with the hypothesis that PRNPB horses represent the descendants of the original genetic pool, a mix of horses imported from the Iberian Peninsula and elsewhere in Europe. Some of the original founders of PRNRB population must have carried the “gait-keeper” *DMRT3* allele upon arrival to the island. From this admixture, the desired traits were selected by the local people over the span of centuries. We propose that the frequency of the mutant “gait-keeper” allele originally increased in the local horses due to the selection for the smooth ride and other characters, long before the PRPF breed was established. To support this hypothesis, we demonstrate that PRNPB horses, and not the purebred PRPF, carry a signature of selection in the genomic region containing the *DMRT3* locus to this day. The lack of the detectable signature of selection associated with the *DMRT3* in the PRPF would be expected if this native breed was originally derived from the genetic pool of PRNPB horses established earlier and most of the founders already had the mutant allele. Consequently, selection specific to PRPF later focused on allels in other genes (including *CHRM5, CYP2E1, MYH7, SRSF1, PAM, PRN* and others) that have not been previously associated with the prized paso gait phenotype in Puerto Rico or anywhere else.

## Introduction

Since their domestication, horses have been selected for many traits including the ability to perform additional gaits other than the common walk, trot, and gallop. Among these are the ambling gaits, the four-beat gaits are particularly comfortable for the rider. The horse breeds exhibiting them are referred to as gaited horses^1^ and can be found around the globe, suggesting that “gaitedness” is an old trait that was selected independently in many breeds^2^. It is thought that altered gait phenotypes require a specific “gait-keeper” mutation in the *DMRT3* gene that affects the configuration of the spinal circuits controlling stride in vertebrates^3^.

The Puerto Rican Paso Fino (PRPF) is a naturally gaited light horse breed prized for its specific phenotypic characteristics, including smooth, natural, four-beat, lateral ambling gait referred to as “paso”^4^. PRPF has served as an icon of local pride and tradition in Puerto Rico, and its origin is directly connected to the history of the West Indies that were the destination for immigrants and their livestock in the Caribbean since the initial arrival of the Spanish settlers until the conquest of Mexico. During this time, various Iberian horses likely related to the modern Spanish Jennet, Barb, Andalusian, Lusitano, and Sevillian Jacas, as well as northern Spanish breeds including Celtic ponies^5^, were introduced to Cuba, Hispaniola and Puerto Rico as well as other Caribbean islands^6^. Those imported horses likely represented the genetic variation that existed in their countries of origin, which, in this case, meant Spain^7^.

Puerto Rico (PR) promptly became the breeding ground for horses that were later exported from the island for the Spanish conquests of Mexico, Honduras and Peru. The resulting admixture of the imported breeds on the island eventually resulted in the local mixed variety called, quite literally, “the Criollo”. These horses are small in stature but powerful horses with a variety of gaits and quite capable of carrying big cargo with little effort. Even as traditional uses for horses have drastically declined in the last century, the nonpure bred (NPB) Criollo horses are still ubiquitous on the island of Puerto Rico. While it makes sense to assume that PR NPB horses must have been the original stock from which the prized purebred PRPF ultimately originated, the genetic link between the two has never been established.

The PRPF that arose in Puerto Rican farms were used by the landowners, and their foremen that supervised their plantations on horses, often with their entire families. They selected horses that would walk smoothly and securely on the uneven, slippery, mountainous terrain in the interior of the island. Thus, in addition to the four-beat, rhythmic, lateral ambling gait, the Puerto Rican Paso Fino developed a quick, isometric, short step in which it barely rises its hoof, and with an extensive use of its ergots and fetlocks, lands a soft footstep without any lateral deviation. In their gait, the movement of the hocks is isogonic. Through time, other characteristics were also selected, especially a long torso for a more comfortable ride. By 1840, the term “paso fino” had already been minted for this race, and additional traits with cosmetic purposes were selected for by different breeders; most common among them a thick, abundant mane, a long, elegant tail, and bright, yellow (sometimes called “tiger”) eyes^8^. By the middle of the nineteenth century, the “paso fino” competition events were common in Puerto Rico, suggesting that the PRPF bread was already firmly established and widespread.

In this study, we looked for genetic clues to understand the origins of the famed Puerto Rican breed. We looked for mitochondrial markers in Puerto Rico and at genetic diversity in nuclear markers compared to other horse breeds. We diversity between the purebred PRPF and Puerto Rican Criollo (nonpurebred or NRB) horses to understand the connection between them, paying specific attention to the frequencies of the alleles in the *DMRT3* gene. Finally, we focused on the genetic diversity at the chromosomal level and looked at the distribution of genetic diversity and signatures of selection in the genomic regions containing specific alleles that have been previously reported to be responsible for the prized “gaitedness” trait.

## Results

### Diversity of the mitochondrial DNA haplogroups

**Figure 1:**
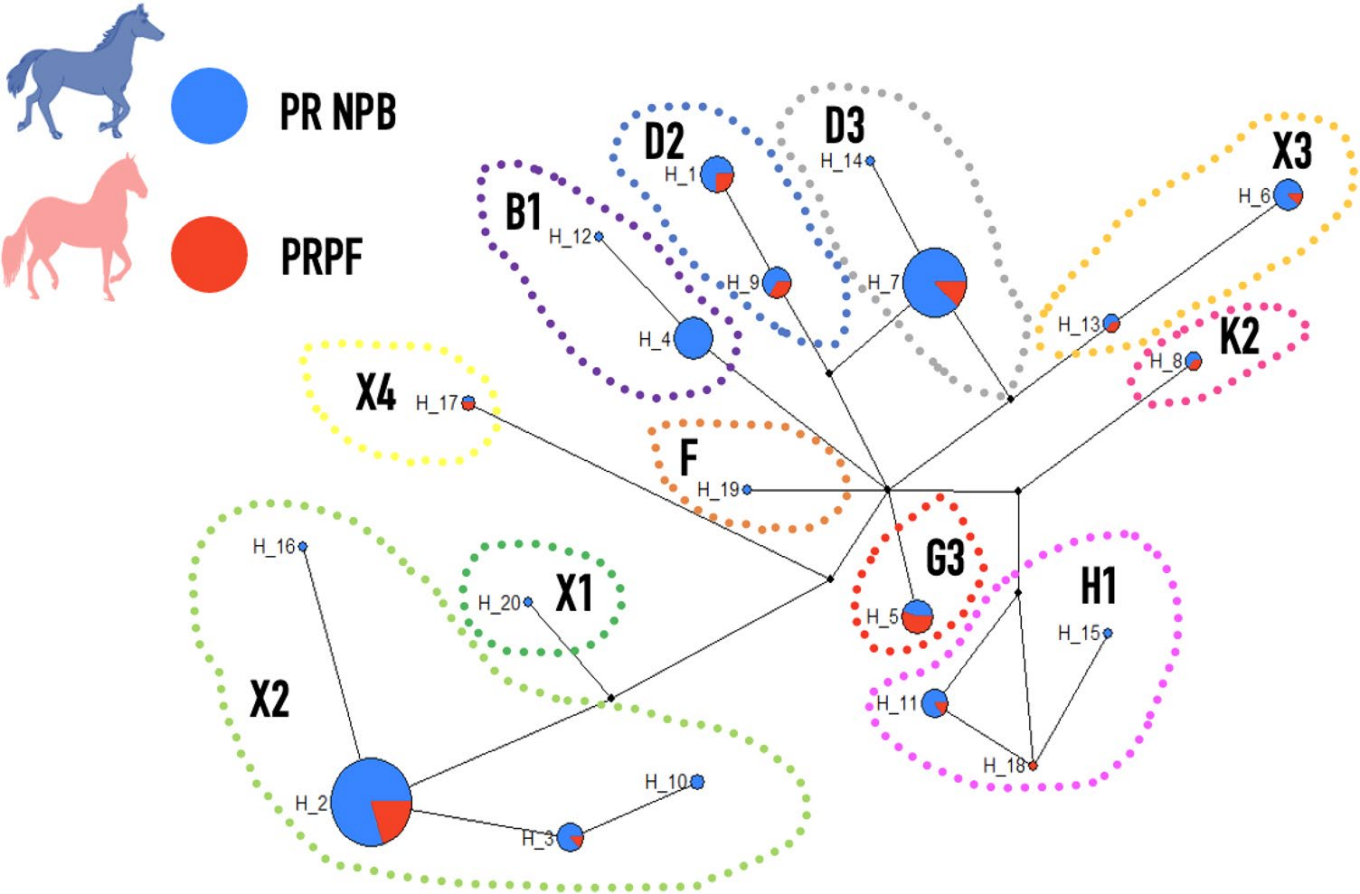
Median Joining Network with all haplotypes in the study. The haplotypes are grouped according to the known haplogroups used in Cieslak et al.^10^. The area of each circle is proportional to number of times each haplotype is present. Small black circles indicate missing haplotypes inferred by Network 5 (Flexus-Engineering, Clare, Suffolk, UK)^9^ . The red color inside the circles represents the proportion of the PRPF samples, while the blue represent PR NPB. Additional details are listed in Table S1.

To establish the relationships between the two Puerto Rican breeds, we collected samples across the island and sequenced D-loops in 200 horses using Network 5^9^ (Fig. 1). With these data, we reconstructed mitochondrial haplotypes and built a haplotype network to see if there were any breed-specific lineages separating them. A total of 20 haplotypes with 24 polymorphic sites were found in 162 PRNPB and 38 PRPF, which included 23 transitions and one transversion compared to the most common haplotype (Hap_1, Table S1). Many of these haplotype sequences matched those previously reported in the literature and had cross-referenced them to the nomenclature used in Cieslak et al.^10^. However, some of the haplotypes reported carry unique mutations (Table S1).

Some of the haplotypes were more common than others. For instance, 20 horses carried haplotype Hap1 (Table S1), which is the same haplotype as the horse reference sequence X79547^11^ and was designated here as Hap1 (Table S1). Hap9 was the most closely related to the reference with a single polymorphic site (15,495 C), while Hap2 and Hap16 differed the most, with 8 polymorphic sites each (Table S1). Among the identified haplotypes, the one denominated as Hap2 (one of the X2 haplogroups) was the most frequent, with 34.17%, followed by Hap7 (haplogroup D3), with 20.6%. We used previously pubished reference haplogroup designations to make our dataset compatible with other sites focusing on horse mitochondrial sequences^10^. The combined frequencies of haplotypes that belong to haplogroups X2, D3, and D2 were the most common, observed at overall (combined for PRPF and NRB) frequencies of 40.7%, 21% and 10.1%, respectively. All other haplotype and haplogroup frequencies are shown for the PRPF and PRNPB samples in Table S1. While the nucleotide diversity of mtDNA sequences showed similar values between PRPF and NPB horses (0.023 ± 0.069 vs. 0.025 ± 0.036), the haplotype diversity in PRNPB was slightly higher (0.825 ± 0.155 vs 0.907 ± 0.199). The main purpose of the haplotype network analysis was to show that there was no breed-specific structure between mitochondrial sequences in our samples (Fig. 1). The complete absence of specific maternal lineages dedicated to the PRPF, supports the close genetic connection between the two breeds on the island.

We further surveyed the frequencies of each haplotype in PRPF and PRNRB samples. Among these, some haplotypes belonged to the same reference haplogroups (Ex. Haplotypes 2, 3, 10, and 16 are all defined by a single X2 haplogroup). Other haplotypes, such as Hap8 and Hap15, did not exactly match earlier described haplogroups but had defining motifs that allowed them to be assigned to haplogroups K2 and H1, respectively. Among the 20 haplotypes found, only one haplotype (Hap18) assigned to haplogroup H1 was unique to the PRPF, while 8 haplotypes were unique to the PRNPB (Fig. 1, Table S1). The majority of the haplotypes (19) could be found among the PRNPB, while only 12 haplotypes were found among the PRPF, and 11 of these were also shared with the PRNPB. In other words, the maternal lineages of the two breeds are largely the same.

### Genome diversity, structure and admixture

**Figure 2:**
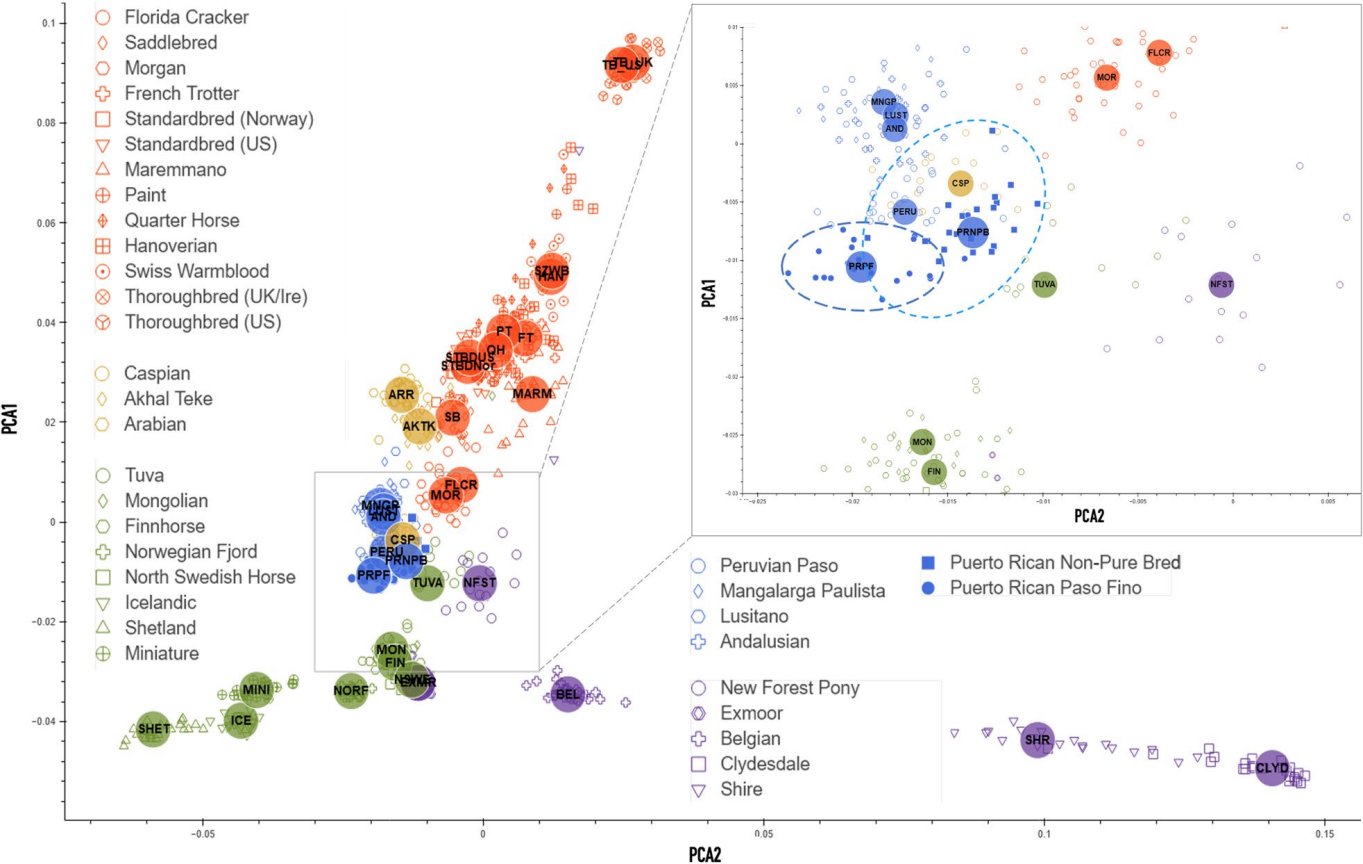
Principal component analysis (PCA) plot of horse breeds incorporated from Petersen et al.^13^, including Puerto Rican Paso Fino (PRPF) and Puerto Rican Criollo (PRNPB) from this study (both are encircled in the enlarged area). For convenience, the horse breeds are colored according to the phylogeny calculated from SNP frequencies in 38 horse populations from Petersen et al. (2013) (Fig. S2). The index for the abbreviations used here is presented in Fig. S2.

The genome-wide diversity of the two horse breeds was evaluated with the Illumina Neogen Equine Community Array containing 65,157 markers^12^. Then, using an overlap between genome-wide genotyping arrays, we increased our sample set of PRPFs by adding publicly available data from a broader study based on similar technology^13^. The two largest components (PC1 and PC2) from the principal components analysis (PCA) were then matched to all horse samples across of the studies to separate the Puerto Rican horses from other American breeds. The resulting distribution shows genetic differences between Puerto Rican horses and all other breeds (Fig. 2), which can be clearly distinguished: the two breeds (PRPF and PRNPB) form a distinct spread along a “vector” (shown by the oval in the magnified insert in Fig. 2), with PRNPB occupying the proximal region and PRPF at the distal region of the spread. The three Iberian breeds that occupy the same branch on the phylogenetic tree (Lusitano, Andalusian and Mangalanga Paulista, group “C”, Fig. S1) are clustered nearby, as expected but, horses from other branches seem to cluster even closer, specifically, the Caspian and Tuvan horses. While some of the diversity in the nonpurebred NPB Puerto Rican Criollo horses is also shared with the Peruvian Paso, the PRPF horse genotypes form a clearly distinct cluster, distinctive from all other breeds (Fig. 2).

**Figure 3:**
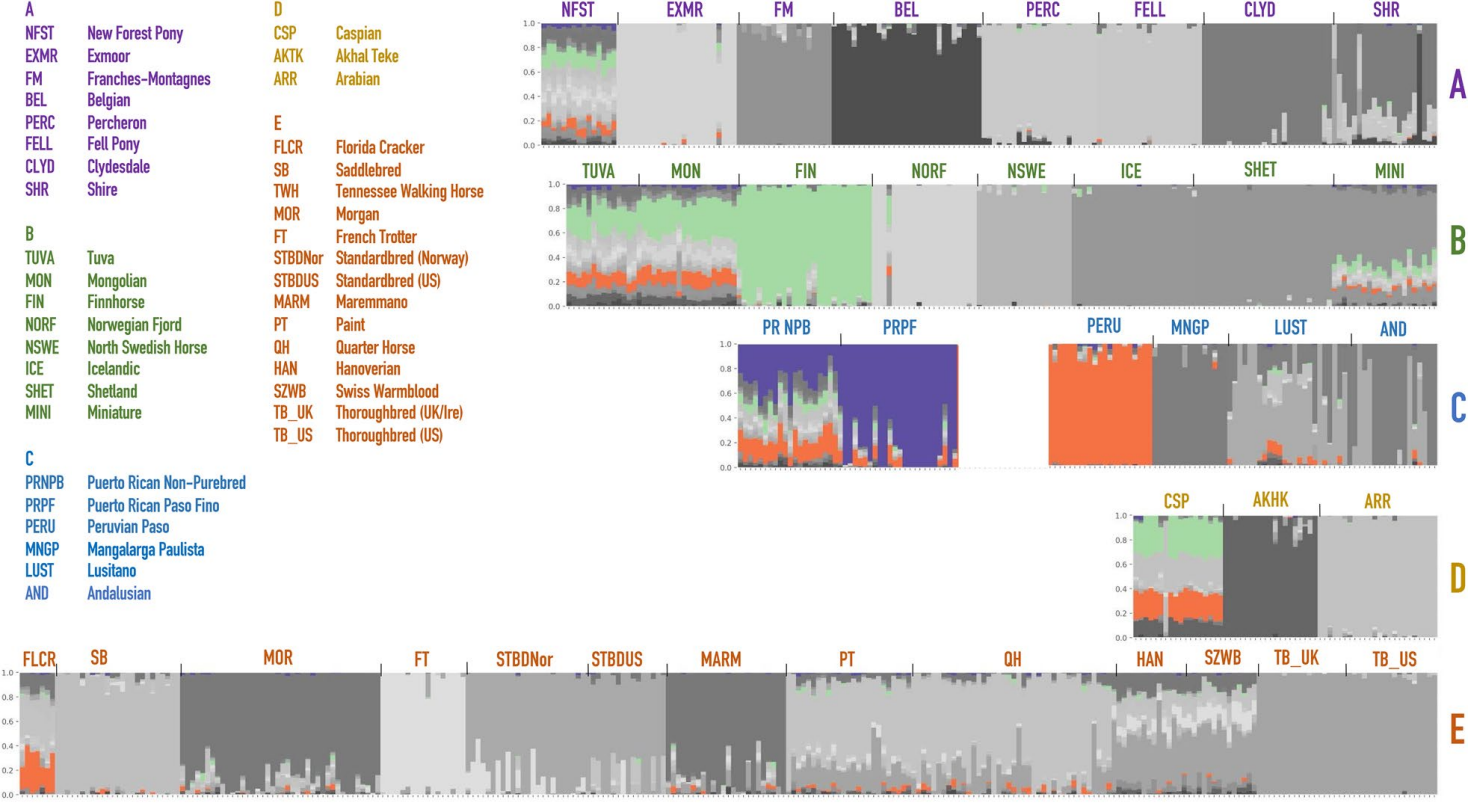
Admixture graph of horse breeds masking components that are very rare in the Puerto Rican Non Purebred (PRNPB) Criollo horses (< 1% of population structure, in gray). As a result, the population components that may have been shared are clearly visible. Data on PRPF and Criollo horses are from the Illumina Neogen Equine Community Array genotyped in this study, and other genotypes are from Petersen et al.^13^. The admixture components that were very rare (< 1%) or absent in PRPF or PRNRB were discolored by the *MixPainer* tool^14^. Capital letters indicate groups highlighted in Fig. 2 and Fig. S2. An admixture plot of horse breeds masking components that are rare (< 5%) in the NPB *Criollo* horses, as well as the unmasked plot, is presented in Fig. S3 and Fig. S4.

The admixture analysis performed on the merged datasets identified genetic components that may be shared between the two Puerto Rican horses and other breeds. While the original visualization of all horse breeds produceed a hard to read cluttered plot, we highlighted segments^14^ that were shared between different breeds (Fig. 3). For convenience, the component colors were preserved from the structure analysis in earlier studies^13^, and the new plot was separated into five larger groups based on the neighbor-joining tree calculated from SNP frequencies in 38 horse populations in Petersen et al.^13^ (Fig. S1). According to the structure and admixture analysis, Puerto Rican horses share population structure components with a number of horses worldwide (Fig. 3). In particular, PRNPB horses share genetic diversity components with New Forest Pony, Tuva, Mongolian and Caspian horses and Florida Cracker. All of these horses show multiple genetic components (shown by the presence of different colors in the plot), which indicate diversity within the breeds. On the other hand, the PRPF genome is largely dominated by a single (purple) component that seems to be almost entirely unique to this breed. However, traces of this component can also be seen in New Forest Pony, Tuva, and Mongolian horses (Fig. 3). Interestingly, whie the Peruvian Paso also shares a genetic component with PRNPB, it seems to be dominated by a different (orange), more common component than that which is prevalent in the PRPF.

### Frequency of the gait-keeper mutation and signatures of selection

We genotyped all 200 horse samples available to us for the presence of the “gait-keeper” *DMRT3* mutant allele previously associated with the paso gait and combined them with the information available on other horse breeds from^1^. Table 1 shows the frequency of the mutant allele included for comparison with other breeds (modified from Promevorá et al.^1^). Remarkably, the frequency of the mutant allele in the PRNPB population was very high (87.4%, Table 1). This is unexpected because paso gait has never been reported in the literature as the phenotypic character for the PRNPB. This finding has prompted us to inquire into the possible history of selection and the relationship between the two Puerto Rican breeds.

**Table 1:** Genotype and allele frequencies of the *DMRT3* Ser301STOP mutation (A allele) in Puerto Rican horses compared to other horse breeds.

Source/Horse Breed Name	Origin	n	A/A	C/A	C/C	A allele (%)	*X*^2^	*p*-val	Gaitedness
**This study**									
Puerto Rico Paso Fino (PRPF)^★^	Puerto Rico	37	37	0	0	100.0	–	N/A	*Gaited—paso*
Puerto Rican Nonpurebreds (NPB)^★^	Puerto Rico	143	108	34	1	87.4	0.93	N.S	*Some/unknown*
**Central and South American Horses** *									
American Paso Fino	USA	34	31	3	0	95.6	0.07	N.S	*Gaited—paso*
Puerto Rican Paso Fino ^★^	Puerto Rico	78	77	1	0	99.4	0.00	N/A	*Gaited—paso*
Brazilian Criollo	Brazil	21	0	1	20	2.4	0.01	N.S	*Some—trocha*
Colombian Paso	Colombia	80	75	1	4	94.4	62.27	< 0.001	*Gaited—paso*
Colombian Criollo	Colombia	35	1	8	26	14.3	0.16	N.S	*Unknown*
Colombian Trocha Pura	Colombia	67	2	10	55	10.4	2.74	N.S	*Some—trocha*
Colombian Criollo Trocha y Galope	Colombia	4	0	2	2	25.0	0.44	N.S	*Some—trocha*
Mangalarga Paulista ^★^	Brazil	14	0	2	12	7.1	0.08	N.S	*Some—marcha*
Peruvian Paso	Peru	22	22	0	2	100.0	24.00	< 0.001	*Gaited—paso*
Venezuelan Criollo	Venezuela	21	0	7	14	16.7	0.84	N.S	*Some*
**Iberian Horses** *									
Asturcon	Spain	24	0	0	24	0.0	-	N/A	*Some*
Barb	North Africa	15	0	0	15	0.0	-	N/A	*Not gaited*
Lusitano^★^	Portugal	19	0	0	19	0.0	-	N/A	*Not gaited*
Losino	Spain	10	0	0	10	0.0	-	N/A	*Not gaited*
Potoka	Spain	10	0	0	10	0.0	-	N/A	*Not gaited*
Pura Raza Gallega	Spain	3	1	1	1	50.0	0.33	N.S	*Some*
Retuertas	Spain	10	0	2	8	10.0	-	N/A	*Unknown*
Sorraia^★^	Portugal	16	0	0	16	0.0	-	N/A	*Not gaited*

Information on Central, South American and Iberian horses is included for comparison (modified from Promevorá et al.^1^).

*Frequencies from Promevorá et al. (2014).

^★^Horse breeds with whole genome genotypes.

**Figure 4:**
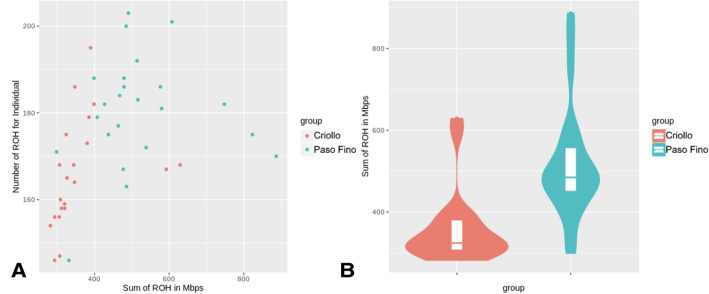
Runs of homozygosity (RoH) in the genomes of the two Puerto Rican horse breeds. (**A**) Number vs sum of length included in the RoH per individual in PRPF vs PRNPB (Criollo) horses. (**B**) A significant difference in the distribution of ROH extent (sum of length) between PRNPB (Criollo, red) and PRPF (Paso Fino, teal) horses.

The PRPF horses showed higher levels of inbreeding than the nonpurebred Criollo horses, with a coefficient of inbreeding Fmax = 0.23 for PRPF compared to F max = 0.16 for the NRB Criollo horses. The SROH regions are also more numerous in Paso Fino than in Criollo horses (average in PRPF vs average in PRNPB, Fig. 4), and these are almost three times larger in size (average in PRPF vs average in PRNPB, Fig. 4). This would be expected if PRPF founders were originally selected from the PRNPB genetic pool. Since all PRPF are gated and PRNPB were never previously reported as gated horses, we expected to see selection for the DMRT3_Ser301STOP mutation in PRPF. Therefore, we performed a genome-wide analysis for extended haplotype homozygosity (EHH) using his statistics^15^ across all the Puerto Rican samples in our study as well as in those breeds where genome-wide genotyping data were publicly available (Table 1).

**Figure 5:**
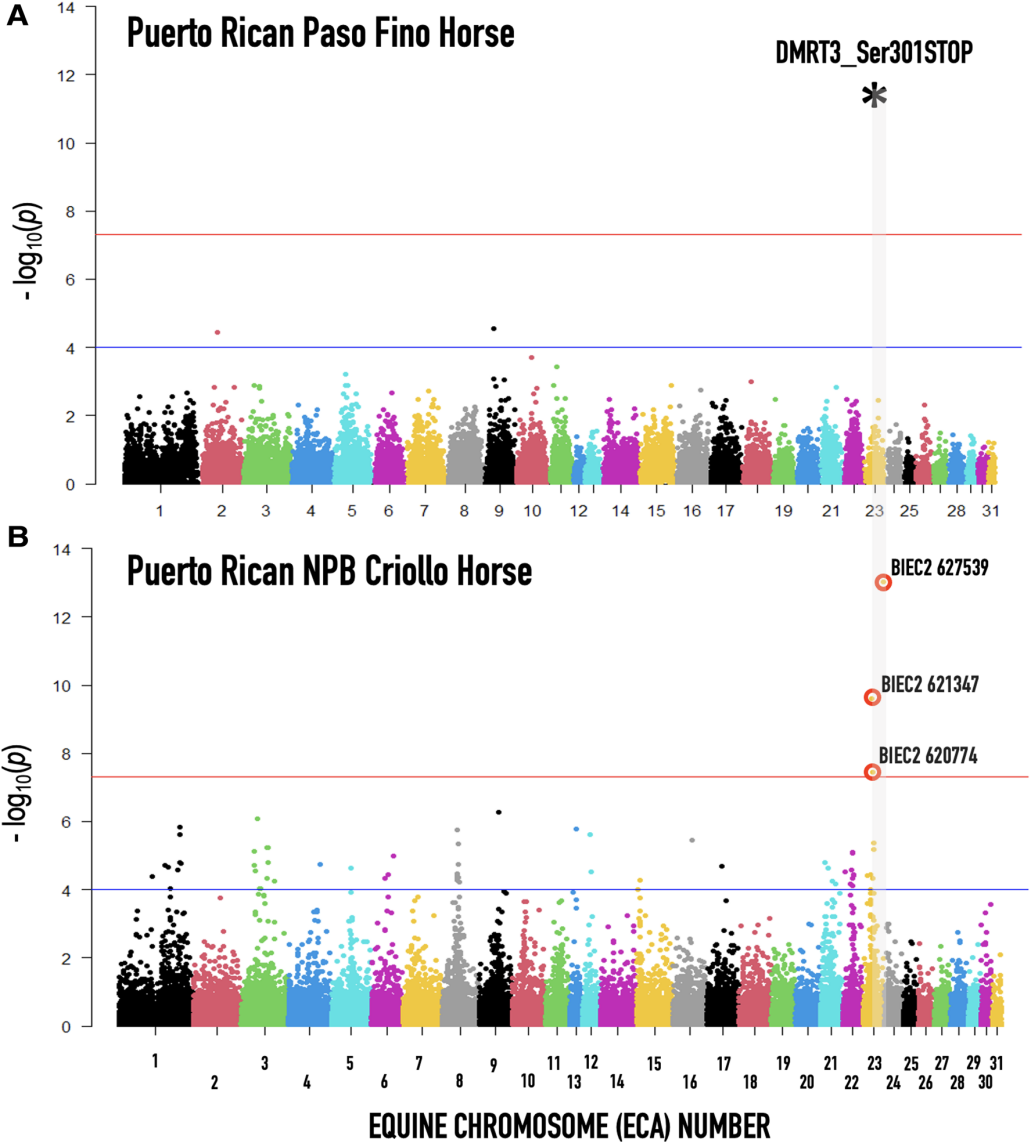
Integrated extended haplotype homozygosity (iHs) for (**A**) Puerto Rican Paso Fino (PRPF) and (**B**) Puerto Rican Criollo (PRNPB) horse. The blue line represents SNPs showing recent selection signatures corrected for the multiple testing significant at the chromosome level, with red line cutoff—SNPs significant at the whole genome level. The shaded area indicates the location and the region of our marker of interest *DMRT3*_Ser301STOP mutation (BIEC2 620109, marked by an asterisk).

The analysis shows a strong genome-wide selection signature in the PRNPB horses (Fig. 5). The location of the *DMRT3*_Ser301STOP mutation (BIEC2 620,109, marked by an asterisk (*) in Fig. 5) showed some indication for the presence of a signature of selection (p < 0.05), but it was directly adjacent to three other markers (BIEC2 627539, BIEC2 621347, and BIEC2 620774) that showed the highest significance level in the genome adjusted for multiple testing (above the red line). Since genomes of domestic horses, including PRPF, are highly homozygous and because this method discards alleles with minor allele frequency < 0.05^15^, this finding had to be verified in the specific genomic neighborhood of the candidate mutation. Therefore, we focused our attention on the variation in SNPs located in close proximity (± 2 Mb) from the *DMRT3*_Ser301STOP mutation on ECA23 of the PRNPB and PRPF horses using *rehh 2.0*^16^.

**Figure 6:**
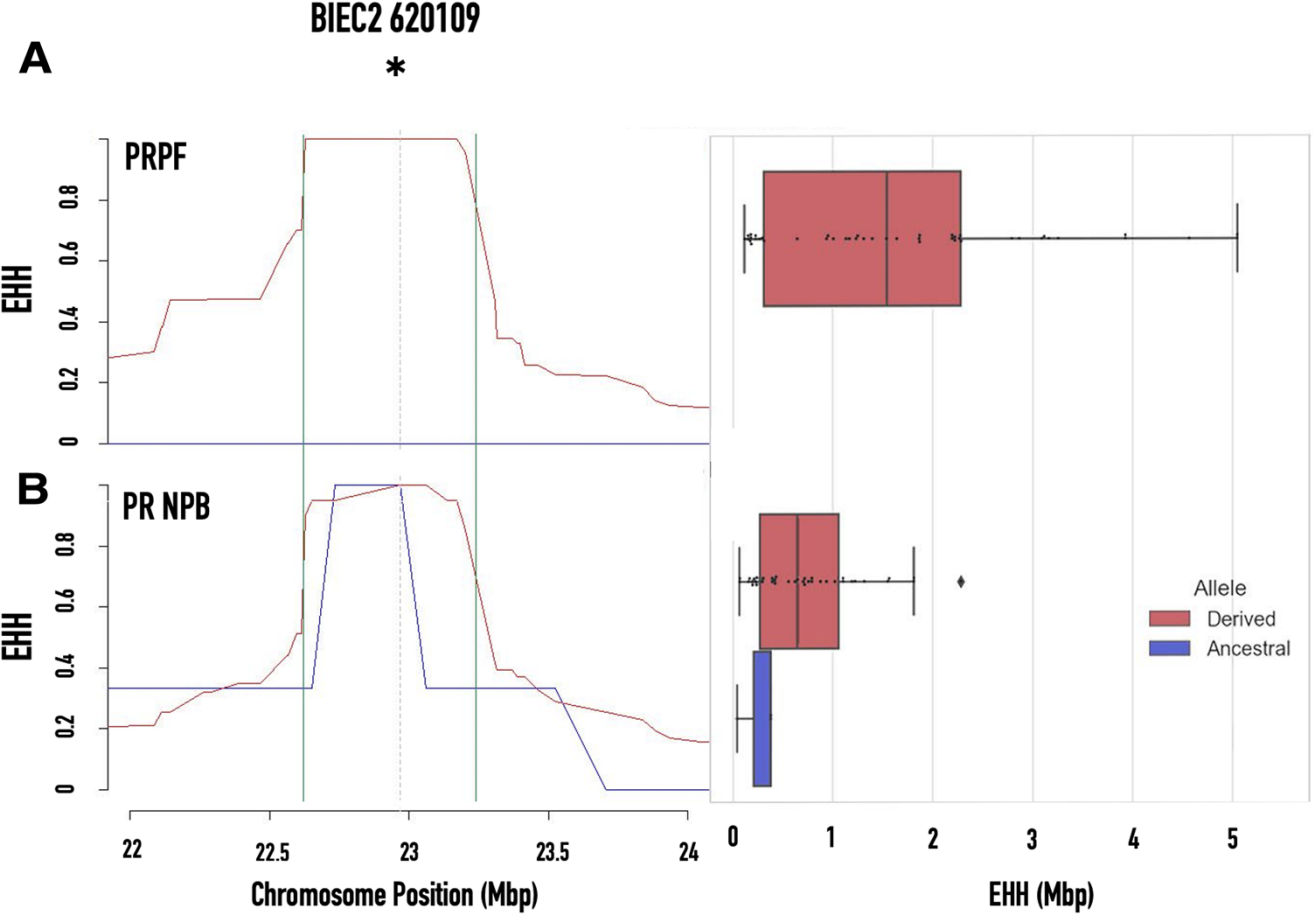
Extended haplotype homozygosity (EHH) decay graph (left) and shared haplotype length graph (right) for the regions around the *DMRT3*_Ser301STOP mutation on chromosome 23 associated with the paso gait (locus BIEC2 620109). The extended homozygosity is more pronounced for the haplotypes containing the nonsense (**A**) allele (derived, red line) in gaited horses from Puerto Rico: (**A**) PRPF (**B**) PRNPB horses but decays quickly in the haplotypes containing the alternative ancestral allele (blue line, ancestral).

The decay of selection around loci BIEC2 627539, BIEC2 621347, and BIEC2 620774, shows the highest significance level in the genome adjusted for multiple testing from Fig. 5, is shown in Fig. 6 and Fig. S5. Homozygosity decay upstream and downstream from *DMRT3*_Ser301STOP (BIEC2-620109 locus) seems to extend further along the chromosome for the haplotypes containing the gait-keeper A allele than in those containing the ancestral allele (Fig. 6; blue line) in Puerto Rican NPB Criollo horses. While in PRPF horses the ancestral allele is missing, the EHH around the derived allele is extended even further than in PRNPB horses, suggesting that selection on this locus may have taken place in the past.

**Figure 7:**
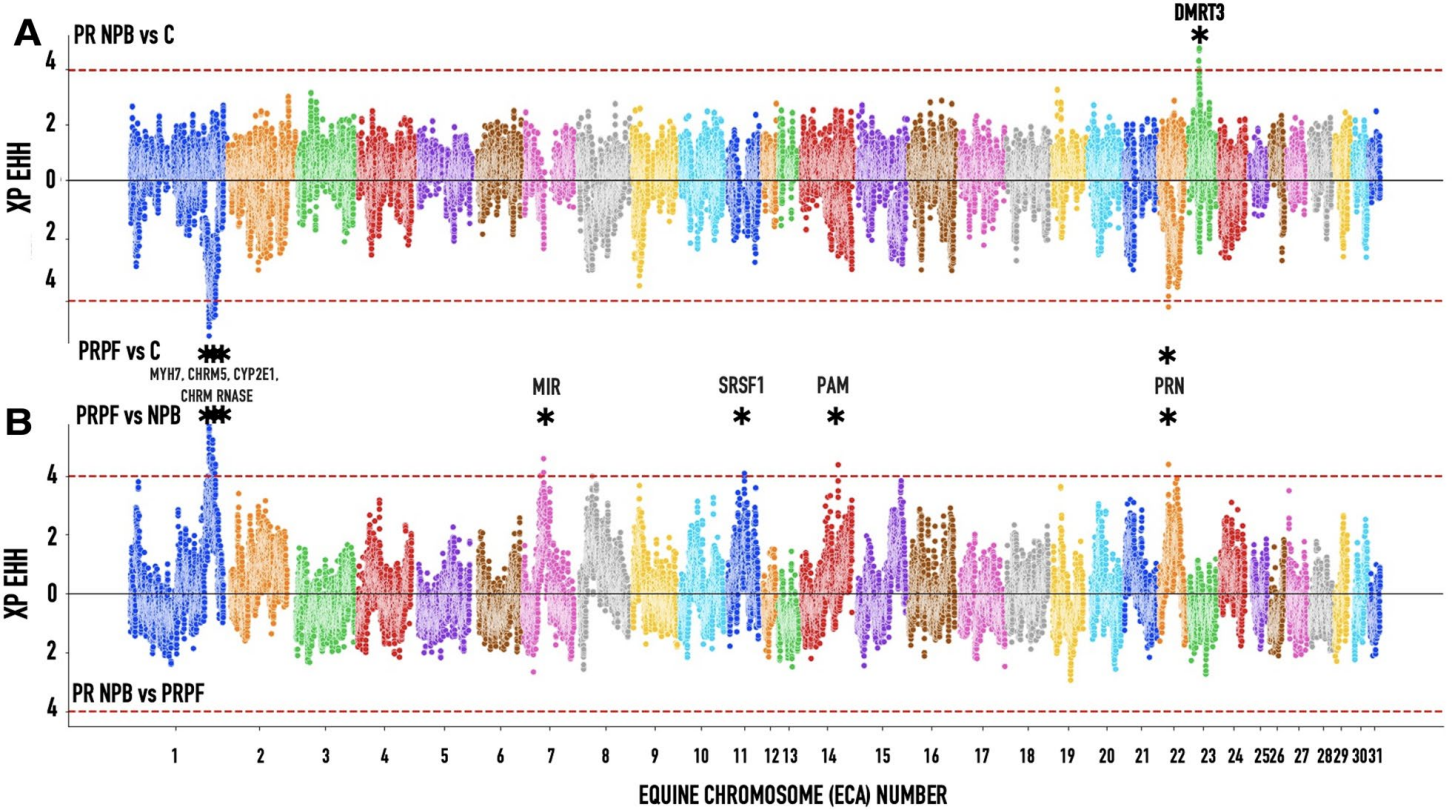
Signatures of selection in Puerto Rico Paso Fino (PRPF) and the nonpurebred (PRNPB) horse genomes based on XP EHH scans of the combined samples in this study. (**A**) Selection tests comparing PRPF and PRNPB horse genomes to the rest of the breeds in the “C” clade (Lusitano, Andalusian and Mangalanga Paulista; Fig. S2). (**B**) Comparing PRPF to PRNPB. Genes with XP EHH values > 4 are displayed on the graph. The detailed comparisons are presented in Fig. S6. The locations of the selection regions are listed in Table S1.

**Figure 8:**
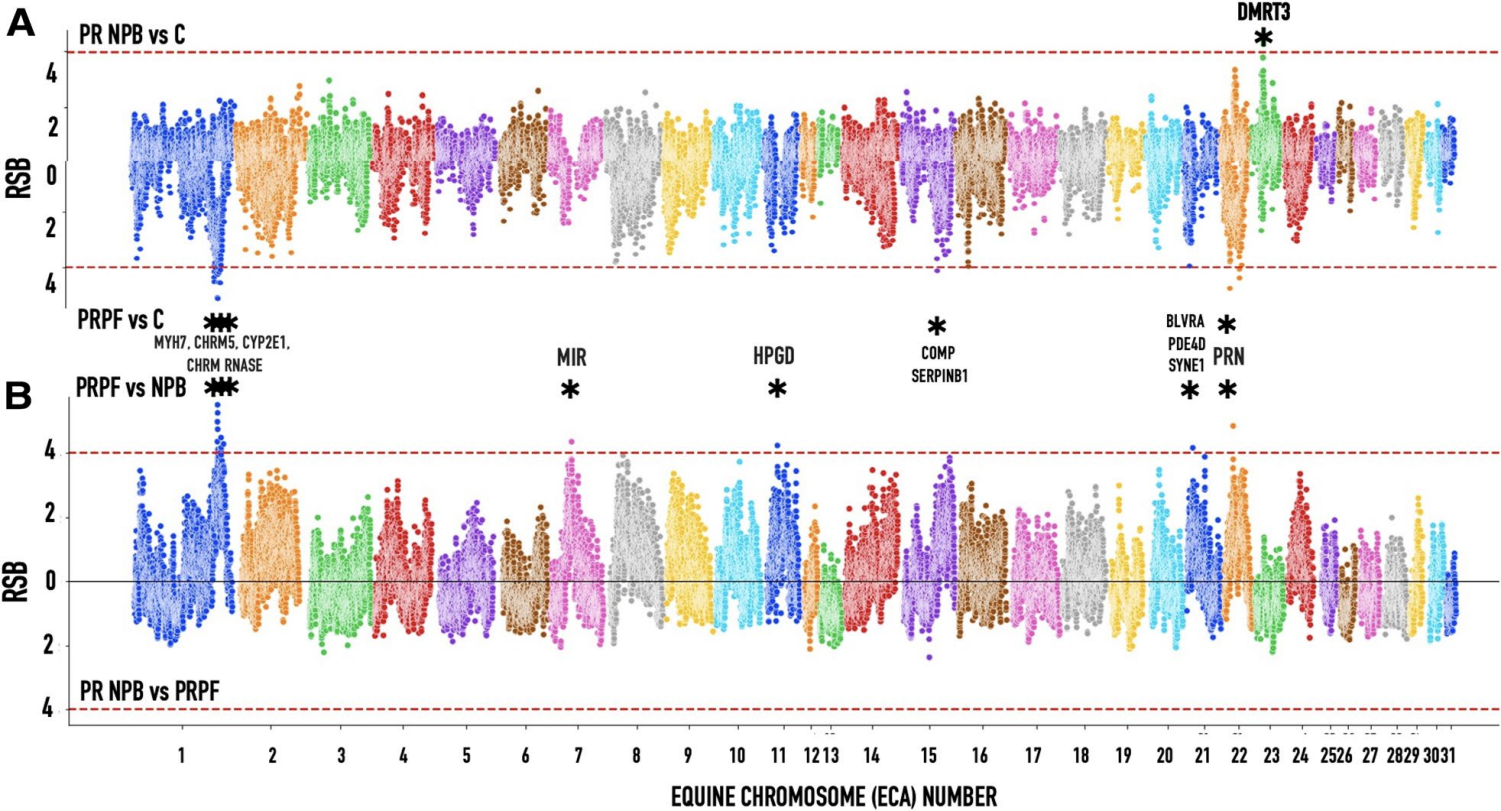
Signatures of selection in Puerto Rico Paso Fino (PRPF) and PR nonpurebred (PRNPB) horse genomes based on the ratio of EHHs between populations (RSB) scans of the combined samples in this study. (**A**) Selection tests comparing PRPF and PRNPB horse genomes to the rest of the breeds in the C clade (Lusitano, Andalusian and Mangalanga Paulista; Fig. S4). (**B**). Comparing PRPF to PRNPB. Genes with RSB values > 4 are displayed on the graph. The detailed comparisons are presented in Fig. S7. The locations of the selection regions are listed in Table S1.

Using genome-wide XP-EHH and RSB tests, we detected strong signature selection in PRNRB horses compared to trees of other breeds (Figs. 7 and 8). In addition to comparing haplotype extent and divergence between PRPF and PRNPB, we also looked for selection signatures by comparing PRPF and PRNPB horse genomes to the rest of the breeds in the clade “C” (Lusitano, Andalusian and Mangalanga Paulista) (Figs. S5 and S6). Genes with XP EHH values > 4 were considered to have a strong signature and are displayed on the graph very close to the DMRT3 gene. The locations of the putative selection regions are listed in Table S2. Neither of the tests showed any selection signatures in the PRNRB compared to the PRPF genomes (Figs. 7 and 8).

## Discussion

Horses have been considered one of our most prized possessions, used for travel, work, food, and pleasure for at least five and a half millennia^17–20^. Nevertheless, the ancestry of various horse breeds and their characteristic traits remains unclear^21^. In this paper, we describe the patterns and the origins of genetic diversity in nuclear and mitochondrial markers and examine the distribution of specific gait-keeper alleles that have been reported to be responsible for the prized phenotype in two Puerto Rican horses: the purebred paso finos (PRPF) and the nonpurebred PRNPB (Criollo).

Over the centuries, the two breeds have gained distinctive appearance that is reflected in the genetic structure revealed by our analysis. We have shown that the PRPF and PRNPB horses are distinct from other breeds (Fig. 2) but nevertheless related as their maternal lineages are intertwined (Fig. 1). We also demonstrated that the “gait-keeper” mutation is almost as common in PRNPB as it is in PRPF. This was surprising, as no selection for the paso gait phenotype has been previously reported for the PRNRB horses. This observation has led us to explore a possible scenario is that PRPF were not originally selected for the paso gait, but picked from the population of local nonpurebred horses (PRNPB), where the “gait-keeper” mutation was already established either by the founder effect or by centuries of selective breeding by the local farmers.

To gain further insight into the origin of the two breeds, we used modified structure plots^22–24^ and developed our own tools^14^ to look at genome contributions in the context of population variation among the worldwide breeds^13^. While we cannot clearly identify distinct sources of genomic admixture in the Puerto Rican breeds, the PCA clearly supports the notion that PRPF is a distinct and native breed among the American Horses of Iberian origin, most closely related to the PRNPB (Criollo) population, but completely distinct from all other breeds (Fig. 3). One likely interpretation of these results is that the PRPF founders were originally selected from the pool of the admixed horses on the island of Puerto Rico, represented today by the PRNPB, as over the centuries the local farmers selected individuals based on the desired phenotypes, especially the gait.

The selection for the desired phenotypes over the centuries should have left its mark on the horse genomes. However it is not easy detect: our genome-wide analysis of genome variation indicates that both Puerto Rican horses have high levels of inbreeding which are comparable to those of many other horse breeds. While each population shows extensive homozygosity regions devoid (ROH), there are differences in magnitude, indicating differences in population histories (Fig. 5). At the same time, even the purebred PRPF is more outbred than roughly half of the horse breeds surveyed in Petersen et al.^13^, and the PRNPB horses of Puerto Rico are even more outbred.

Due to the maternal inheritance of mtDNA and lack of recombination, mtDNA has been widely used for studying the history of maternal lines. Mitochondrial DNA, specifically its control region, has been used effectively to study the origin and diversification of domestic horses worldwide^6,7,17^. Over the years, various studies have proposed that the variability found in the mtDNA of horses can be traced and restricted to geographic regions^10,25,26^. One of the first to propose such a hypothesis discovered 17 frequent haplotypes (mtDNA sequences), each creating a distinctive cluster. We used mitochondrial D-loop sequences from our samples for the preliminary assessment of the origin of the domestic horses on the island, specifically using hypervariable region 1 (HVR1) in a cost-effective approach to help understand the origins and diversification of the Puerto Rican horses. The sequences were grouped into 54 unique sequences (haplotypes) that could be cross-referenced to the nomenclature used in Cieslak et al.^10^.

Recent studies using mtDNA confirmed Iberia as the geographic and genetic source for the New World horses, as several predomestic maternal lineages unique to the peninsula still survive in modern horses of Iberian descent^27^. Generally, these breeds were established by the haplotypes that came from multiple sources, but the frequency of Iberian haplotypes in New World breeds is generally consistent with the historical documentation of their origins^7^. Specifically, haplogroup D, as defined by Jansen et al.^25^ and later redefined by Cieslak et al.^10^ as haplogroup X, is well represented in both the Southern Iberian and New World breeds, thus suggesting the importance of Iberian breeds in founding horse varieties of the New World^5,7^.

Our study of the mtDNA diversity in the two Puerto Rican horses also points to the mainly Iberian origins, since haplotypes D and X are the ones most represented (Fig. 1; Table S1). There seem to be many shared haplotypes among the two breeds in Puerto Rico: among the total of 20 haplotypes found, 19 were identified among the PRNPB, and out of 19 haplotypes found in PRNPB, 11 were also shared with the PRPF, which in turn had only a single unique mtDNA haplotype (Fig. 1). This particular haplotype is most likely to have been missed in the PRNPB population due to the limited sample size and may be encountered with more extensive sample selection, as it belongs to the haplogroup H1 found in other Iberian and New World horses and differs only in two positions with Hap15. This is consistent with the scenario where the ancestral pool was formed from many Iberian breeds arriving at the island and establishing the original genetic pool.

The direct inheritance from mother to daughter without recombination can provide valuable clues in the preliminary assessment of ancestry in the maternal lineages that can be used in reconstructing the history of the breed’s origin. Since previous research has shown at least some genetic clustering of haplotypes, mtDNA analysis allows us to make a preliminary assessment of maternal lineages^7^. However, there is a high level of variability within and among horse breeds, without a clearly defined geographical pattern of distribution^28^, so the mtDNA evidence alone is not sufficient to fully describe the ancestry of the Puerto Rican breeds. Therefore, the identification of the population origin required a more complex genetic approach that included dense genotyping across the genome.

Thanks to the analysis of the genome-wide array data, we can see that Puerto Rican horses share genome variation components with a number of horses worldwide (Fig. 3). In particular, the PRNPB horses appear to have genomic fragments in common with the Northern European and Asian horse breeds (Fig. 3, top row). Specifically, they share the “light green” and the “orange” components with the Finnhorses, Mongolian and Tuvan breeds. This appears to be the same component present in the Iberian (Lusitano), Middle Eastern (Caspian horse), or US derivatives from the Spanish stock brought to Florida in the 1500s (Florida Cracker). The “orange” component present on the island, also completely dominates the Peruvian Paso, the breed that is most closely related to the PRNPB horses outside of Puerto Rico. Both Puerto Rican breeds display a common “purple” component that seems to be unique to the local island horses and cannot be found in any of the surveyed horse breeds at the time (Fig. 3). This component represents a larger part of genetic variation in the PRNPB horse (which also has green and orange components shared with other breeds) but completely dominates the PRPF genomes. The most likely explanation of this observation is that the PRNPB horse has a unique mixture that incorporates variation from a diverse set of lines brought on the ships to the island, and the PRPF has been selected for this particular set of variation from the admixed pool. If the latter statement is true, PRPF should have less genetic diversity than PRNPB.

We observed extended runs of heterozygosity (ROH), contiguous uninterrupted stretches of chromosomes without any heterozygous SNPs^29^, which may be a consequence of natural or artificial selection on genome-wide variation, as selection for one allele would have swept variation across the linked loci^30^. In fact, ROH approach is commonly used to test hypotheses for artificial selection in domesticated animals^31,32^. The observed differences in ROH are indeed consistent with the hypothesis that PRPF has been under selection (Fig. 4). However, the extended ROHs are not a definite indication of recent artificial selection, as they can be derived from consanguineous mating in a small population (i.e., drift). Therefore, it is important to distinguish the signature of selection around the targeted locus from the signal of inbreeding across the entire genome. A good candidate for this analysis is the “gait-keeper” mutation in the *DMRT3* gene with a known major effect on altered gait characteristics, such as the paso gait of the PRPF^1,3^. Nevertheless, the large regions of homozygosity spanning across portions of entire chromosomes (Fig. 3) in these horses make selection tests based on population variation difficult to use^30^.

The “gait-keeper” *DMRT3* mutant allele (allele A, Ser301STOP) shows high frequency in many gaited breeds and breeds bred for harness racing, while other horse breeds were homozygous for the wild-type allele (allele C) in earlier studies^1^. It has also been reported at high frequencies in Northern European breeds (Table 1). For instance, it appears that selective breeding for lateral gaits in the Icelandic horse population could lead to the complete loss of the C-allele^33^. This mutation is not common in the Iberian horses and was only reported there once at low frequency in the Pura Raza Galega breed^1^ (Table 1). On the other hand, many horse breeds in the New World have this allele, possibly due to the admixture with other, non-Iberian breeds. The analysis of 152 Colombian Paso horses (most with phenotypic data) demonstrated selection on the *DMRT3* gene can explain differences in horse gait in that breed^34^. On the other hand, a similar analysis in Mangalarga Marchador and the French Trotter horses shows that *DMRT3*, while associated with the trait, may not be the sole locus that controls the gait ability^35,36^.

The frequency of the *DMRT3* mutant allele in the combined PRPF sample from this and the other studies is the highest reported in all animal breeds (Table 1). Remarkably, it was also present in the majority of the PRNPB; 142 out of the 143 genotyped PRNPB horses had at least one *DMRT3* mutant allele (Table 1). This stands out in comparison to the other criollo horses reported in the literature that have a low frequency of the mutant “gait-keeper” allele (ex. Brazilian, Venezuelan and Columbian, Table 1). These breeds also arose from the mixture of different Iberian breeds, including a strong influence of Portuguese breeds. Why is then the PRNPB different?

In theory, alleles can achieve high frequency due to mechanisms different than selection. For example, genetic drift is expected to result in the fixation of most alleles over time or even instantaneously following the founder effect. To argue for the action of recent selection (selective breeding), the genomic neighborhood of the candidate allele must be evaluated in a formal test. Since the selection for this allele should have been pretty recent, not older than the historic horse arrival to Puerto Rico, the selection tests can be evaluated based using the extended haplotype homozygosity (EHH), population differentiation tests, or a combination of both approaches^30^.

Our reasoning was that, if this allele was favored in one or both of the Puerto Rican breeds, it would be associated with long haplotypes at high frequencies (EHH), typically representing recent selection^37^. Somewhat surprisingly, we did not observe any signatures of selection in the PRPF with genome-wide significance (Fig. 5), which means that there was no specific selection for this genetic variant in the pure bread lines. In contrast, in PRNRB horses, there is a clearly selected region located on chromosome 23 located very close to the *DMRT3* locus.

The major limitation of the selection tests based on haplotypes is that they do not perform well in genomes with low genetic diversity (where selected haplotypes are difficult to identify). Therefore, it is not surprising that the iHs test, a recombination-based test that uses only the variation within the specific horse breed, did not identify any selection signatures in PRPF. This would be expected when (a) there is almost no variation in the locus and (b) only a few variable markers exist on chromosome 23, undermining the performance of EHH^30^. A contrast of diversity and divergence would be a better approach with the reasoning that the haplotypes containing selected loci should show more differences between diverging populations compared to the other loci genome wide (see Materials and Methods). This is why we followed XP-EHH and RSB tests that combined EHH statistics with the degree of population differentiation. For the addition of a phylogenetically based outgroup reference in these comparisons, we used a combination of samples from breeds in the same lineage^1^ (Fig. S2).

Using genome-wide XP-EHH and RSB tests, we detected a strong signature selection in PRNRB horses compared to the outgroup composed of trees of other breeds (Figs. 7A, 8A). Once more, this is a single selected region in PRNRB horses and is located on chromosome 23 next to the *DMRT3* locus. Neither of the tests showed any selection signatures in PRNRB compared to PRPF (Figs. 4 and 5).

The addition of population differentiation has helped to identify several targets of selection in the PRPF genome compared to PRNPB and other horse breeds (Table S2, Figs. 7A, 8A). None of these candidate selection loci were located close to the candidate *DMRT3* gene. However, at least some of them could be potential candidates with functions associated with horse gait selected in PRPF. Among these, the strongest signatures are located next to *MYH7* muscle myosin on chromosome 1 and a prion protein *PRNP* on chromosome 22.

The human homolog of the *MYH7* gene is known to be expressed in human ventricles as well as in skeletal muscle tissues rich in slow-twitch type I muscle fibers, where its expression correlates with the contractile velocity of the cardiac muscle and is altered during thyroid hormone depletion and hemodynamic overloading. Mutations in this gene are associated with familial hypertrophic cardiomyopathy, myosin storage myopathy, dilated cardiomyopathy, and Laing early-onset distal myopathy. The *PRN* gene human homolog may play a role in neuronal development and synaptic plasticity and be required for neuronal myelin sheath maintenance. Mutations in this gene have been associated with Creutzfeldt-Jakob disease, kuru, fatal familial insomnia, Gerstmann-Straussler disease, and Huntington disease-like 1. A list of all the selected targets is presented in Table S2, and a more detailed description of these and other genes selected in PRPF is given in Table S3.

These signatures may reflect other characteristics selected for Puerto Rico: a long torso for a more comfortable ride, a thick, abundant mane, a long, elegant tail, and the yellow eyes. In addition to the naïve genome-wide tests for selection described above, a unique character called “tiger-eye”, characterized by a bright yellow, amber, or orange iris, was chosen for the Puerto Rican Paso Fino breeders. A recent study reported that most of the “tiger-eye” horses were either homozygous for either tiger-eye-associated alleles or were compound heterozygotes^8^. We used our data to independently evaluate the presence of a signature of selection around the *SLC24A5* gene in our PRPF lineages. While this analysis cannot be performed directly on our dataset, since the four markers from that study (BIEC2_60719, BIEC2_61330, UKUL310, and BIEC2-61972) were not included in our genotypes, these were located very close (within 0.5 Mb) from a peak on ECA1 (centered on 141,514,807 bp, Figs. 7, 8 and Figs. S7,S8), indicating an instance of nearby selection that occurred between PRNPB and PRPF, as would be expected. Additional genotypes covering the region of the *SLC24A5* gene as well as the phenotype data would be necessary to verify this finding.

In summary, we have shown that the PRPF and PR NPB horses are related, as their mitochondrial sequences are intertwined (Fig. 1). Then we demonstrated that the “gait-keeper” mutation is almost as common in PRNPB as it is in PRPF (Table 1). Somewhat unexpectedly, we did not see any signatures of selection focusing on this gene in PRPF, but a strong signature associated with this gene was found in PR NPB (Figs. 7, 8). Given our current results, we propose that the most likely historic scenario is that PRPF is a distinct horse breed that has been selected from the local nonpurebred horses (PRNPB). The genetic pool of the PRNPB was likely a result of admixture between the horses historically imported to Puerto Rico from Spain and other regions of the Old World. Some of the founders of this pool must have originally brought the “gait-keeper” *DMRT3* mutant allele (allele A, Ser301STOP) with them. Local farmers must have been selectively breeding for the mutant allele, and over several centuries, it has increased in frequency in the nonpurebred population of horses on the island. Consequently, the founders of PRPF were initially picked out from the existing PRNPB pool, but since the *DMRT3* mutant allele was already nearly fixed, the selection in the purebred horses was focused on other genes that may or may not be associated with the paso gait, including *MYH7*, *PRN* and others. In order to further validate our current hypothesis and to identify the specific functional mutations that have been selected by the PRPF breeders, a comprehensive phenotype-genotype analysis based on horse pedigrees and sequencing data from these candidate genes needs to be conducted.

## Methods

### Samples

We collected hair samples from each of 200 unrelated horses from 27 locations grouped in seven sampling sites across the entire island of Puerto Rico (162 PRNPB and 38 PRPF; Fig. S8). In each case, the owners of the horses confirmed the lack of relatedness between the samples and personally provided an informed consent for using their animals in the study. All hair collections were done in the presence and with supervision/help from the horse owner or trainer to avoid any incidents with the animal. All methods were carried out in accordance with relevant guidelines and regulations, and the collection procedure has been approved by the UPRM’s Institutional Animal Care and Use Committee (IACUC)^38^.

The hair samples were collected by plucking at least 15 hairs from each individual with a pair of sterile tweezers in order to ensure follicle extraction. Hair samples were labeled, stored in a plastic bag, and transported to the lab at the University of Puerto Rico at Mayagüez (UPRM) for further analysis. Total genomic DNA was extracted from hair roots (minimum 15 from the same individual horse) using a QIAGEN DNAeasy Blood and Tissue kit following the user-developed protocol 2 (QIAGEN, Germany). Extracted DNA was quantified using an IMPLEN nanophotometer, and stocks of 100 µL at 20 ng/µL for each sample were made for easy access in the future.

While the pedigrees of the PRPF were all well documented, there is no reliable information on ancestry beyond the F1 generation for the PRNPB horses. In each case, the owners of the horses personally confirmed the lack of known relatedness during the collection procedure.

To verify these statements, we calculated the IBD values for all the horses in this study as well as in Petersen et al. study using PLINK^39^ and according to the equations originally developed by Wright^40,41^. No first-degree relatives were detected, but two of the three PRPF horses from our study (OS_PASO) are related at the IBD > 0.271, corresponding to the relationships between the second-degree relatives (Table S4). The OS_PASO horses were not related to any of the horses in the Petersen et al., 2013 study at the level of second-degree relatives, but some of them had pairwise IBD values higher than 0.125, corresponding to the relationships between the third-degree relatives (Tables S4). The average IBD between Paso Fino (PRPF) Paso Fino horses from Petersen et al.^13^ was 0.145 ± 0.004 indicating a moderate amount of inbreeding similar to other breeds (ex. the IBD in the Peruvian Paso in the same study is 0.14 ± 0.002). The highest inbreeding found was between 0.3 < IBD > 0.35 among samples (RP014, RP458, RP499, RP500 and RP504) indicating the level of inbreeding approximately equivalent to half-sibs (Table S5). The values of pairwise IBD proportions between nonpurebred horses from Puerto Rico (PRNPB). The average IBD was 0.02 ± 0.002, indicating low amounts of inbreeding (Table S6).

### Sequencing

The target region of mtDNA containing the D-loop (positions 15,440 to 16,108,668 bp) was amplified with the AGC TCC ACC ATC AAC ACC CAA A (forward) and CCA TGG ACT GAA TAA CAC CTT ATG GTT G (reverse) primer pair^42^ using a 20 µL reaction mix containing 12.5 µL of GoTaq Green Master Mix (Promega USA), 0.5 µL of each primer at 10 µM, 5 µL of genomic DNA, and 2 µL of water. The thermocycler was programmed for 5 min heating at 95 °C, 30 cycles of 40 s at 94 °C, 45 s at 55 °C, 45 s at 72 °C and 10 min of final extension at 72 °C. The resulting PCR product was stained with EtBr and visually inspected on 2% agarose gels. The resulting amplicons were purified with the High Pure PCR Cleanup Micro Kit (Roche, Germany).

Sequencing reactions were performed on an ABI 3730xl DNA Analyzer at EPOCH Life Sciences (Texas USA). The resulting sequences were truncated to a total length of 248 bp (positions 15,494–15,740, part of HVR1) using Geneious 8.1.6 software (Biomatters Ltd.) to make it compatible with the previously published data, and pairwise alignment of forward and reverse sequences was performed for each individual. The consensus sequences were retrieved and aligned to horse mtDNA reference X79547^11^. The haplotypes were also assigned to traditional haplogroups according to Cieslak et al.^10^ and are listed in Table S1.

Sequence files were converted into haplotypes using *DNAsp 5.10*^43^, and median-joining networks were reconstructed using Network 5.0 software (Flexus-Engineering, Clare, Suffolk, UK)^9^. Default settings were applied, and four mutation hot spots were excluded (at positions 15,585, 15,597, 15,650 and 15,604), as recommended in previous studies^10,25,42^. Each haplotype node in the network was assigned a pie chart with a size scaled to represent the number of individuals and color coded (red and blue) to represent the proportions of PRPF and PRNPB for each individual haplotype (Fig. S2). Haplotype diversity and nucleotide diversity were calculated using ARLEQUIN 3.5.2.2 software^44^.

### Genotyping

All 200 samples were genotyped for the *DMRT3* Ser301STOP “gait-keeper” mutation, and genotypes of 180 were recorded. Some PCR reactions failed due to various technical reasons unrelated to the genotype (usually because of the low DNA concentrations or impurities). Since the 90% of the genotypes were recovered, we did not attempt to optimize the PCR reactions.

In addition, we used the Illumina Neogen Equine Community Array (65,157 markers evenly distributed across 31 autosomes)^12,45^ to genotype 24 samples (3 PRPF and 21 PRNPB). Genotypes were obtained from the intensity data using *GenomeStudio* (Illumina) software suite with the minimum score threshold of 0.15. The resulting data were converted to PLINK^39^ format for downstream analysis. We merged the data obtained from our samples with a publicly available dataset of a broader study^13^ that included 795 samples of 37 horse breeds all around the world genotyped on the similar array^13^. After merging, filtering by the genotyping rate and soft pruning the results for LD *(–geno 0.02, –indep-pairwise 50 10 0.2*), a total of 819 samples and 22,347 variants were retained. Notably, the dataset contained genome-wide genotypes of 20 additional PRPF horses that were merged with our data the approximately equal number of genome-wide genotypes from each bread (23 PRPF and 21 PRNPB) included in all the downstream genome-wide analyses.

### Statistical analysis

The entire datasets of the filtered 22,347 markers filtered from the Illumina Neogen Equine Community Array and covering the entire lengths of all the chromosomes (see above) were used for the PCA, Admixture and RoH analysis, as well as in the XP-ESS and RSB selection tests. In the EHH test, the 966 markers on the ECA23 were used to illustrate the haplotype homozygosity around the putatively selected locus.

*Principal component analysis (PCA)* was performed using EIGENSOFT, and the PCA plot was produced using custom Python scripts using libraries for data processing (*pandas, matplotlib, seaborne*)^46^. To reduce the clutter of the visualization for PCA plots, we assigned colors to each breed according to the phylogeny calculated from SNP frequencies in 38 horse populations from Petersen et al.^13^ (Fig. S2). The two largest components (PC1 and PC2) were then used to visualize genetic distances between all horse samples across of the studies. The analysis shows unique genetic makeup of the Puerto Rican horses compared to the other breeds (Figs. 1 and 2).

*The admixture analysis* was performed by ADMIXTURE software^47^ on the data from this study merged with the genomic dataset in Petersen et al.^13^. The optimal number of subpopulations was selected using ADMIXTURE's cross-validation, with k = 25 on 20-fold repetition of the procedure (Fig. S3). To declutter the structure visualization plot that used hundreds of colors corresponding to the distinct admixture components comprising different horse breeds, we designed a custom script to modify the output that masks the components that are rare or absent in the Puerto Rican horses^14^. As a result, we only highlight the components that may have been shared between other horses and the Puerto Rican breeds (Figs. S3, S4). The script can be modified to increase or decrease the threshold for the shared component: alternative versions of the same graph showing all the components (Fig. S3) and only the components < 1% of shared population structure are attached in Fig. S3. For convenience, the component colors were preserved from the structure analysis in earlier studies^13^, and the current plot was separated into five larger groups based on the neighbor-joining tree calculated from SNP frequencies in 38 horse populations in Petersen et al.^13^ (Fig. S2).

*Runs of homozygosity (RoH)* analysis for PRPF and PRNPB was performed using a sliding window approach^48^. Given the low density of SNP coverage, 15 loci were selected as the sliding window size, with a minimum of 5 consecutive SNPs to start the run and a 10 Mbp max distance between SNPs in each run. First, ROH numbers and sizes were calculated separately for every individual and then averaged for each Puerto Rican breed. The coefficient of inbreeding was derived from the RoH analysis^49^. Inbreeding estimates were determined using two methods: individual inbreeding coefficients (Fmax) measure observed versus expected genetic diversity in an individual in a population. The sum of runs of homozygosity (*SROH*) estimates the proportion of the genome covered by fragments lacking any variation in SNPs (heterozygosity).

*Signatures of recent selection* in the two varieties of Puerto Rican horses were evaluated by identifying genomic regions with unusually high local haplotype homozygosity using the *rehh* package for* R*^16^. We integrated variation in all the SNPs from the PRNPB and PRPF horses by combining genome-wide genotypes from this study and^13^. We discarded all the loci that were not present in both studies and phased our genotypes by the SHAPEIT tool^50–53^ using an existing recombination map^54^. The within-population genome-wide analysis “*integrated haplotype homozygosity score*” (*iHS*) was calculated for PRPF and PRNRB as well as other breeds using the *rehh* 2.0 tool described in Tang et al.^15^. In addition, two pairwise population statistics have been calculated comparing the breeds in their phylogenetic context: the *Rsb (ratio of EHHS between populations)* and the *XP-EHH* (cross-population EHH) statistic^37^. We used combined genome-wide genotype data from the three breeds shown to be the most closely related to the Puerto Rican horses^13^, namely, Andalusian, Lusitano, and Mangalarga Paulista (Fig. S2).

To visualize recent selection and contrast haplotypes associated with the selected trait in the genomic region, we used extended haplotype homozygosity (EHH^37^). In this approach, the haplotype frequency and decay of haplotype length were evaluated for the chromosomal neighborhood flanking the candidate marker under selection, for example, the “gait-keeper” mutation BIEC2 620109 (*DMRT3Stop*). The distribution of EHH was tested for other candidate alleles for PRPF and PRNPB vs. reference (i.e., Andalusian, Lusitano, Mangalarga Paulista, etc.; Fig. S5).

## Supplementary Information

Supplementary Information.

## Data Availability

All genotyping data from this project are available from NCBI (pending submission).
