# Supplementary material for: Genetic diversity and selection in Puerto Rican horses
Source: Sci Rep. 2022 Jan 11;12:515. doi: 10.1038/s41598-021-04537-5 (PMC8752667; doi:10.1038/s41598-021-04537-5)
Supplement: Supplementary file 1 — Supplementary Information. [file 41598_2021_4537_MOESM1_ESM.docx]

**SUPPLEMENTARY MATERIALS**

**Genetic Diversity and Selection in Puerto Rican Horses**

**Walter W. Wolfsberger** ^+^**^1,2,3^, Nikole M. Ayala** ^+^**^2^, Antoliy Potapchuk ^2^, Khrystyna Shchubelka ^1,2,3^, Stephanie O. Castro-Marquez ^1,2^, Audrey J. Majeske ^1,2^, Luis Figueroa Oliver ^2^, Alondra Diaz Lameiro ^2^, Juan Carlos Martínez-Cruzado ^2^, Gabriella Lindgren ^4,5^, and Taras K Oleksyk ^*1,2,3^**

^1^ Department of Biological Sciences, Oakland University, Rochester, Michigan;

^2^ Biology Department, University of Puerto Rico at Mayaguez, Mayaguez, Puerto Rico;

^3^ Biology Department, Uzhhorod National University, Uzhhorod, Ukraine;

^4^ Department of Animal Breeding and Genetics, Swedish University of Agricultural Sciences, Uppsala, Sweden;

^5^ Livestock Genetics, Department of Biosystems, KU Leuven, Leuven, Belgium.

* [oleksyk@oakland.edu](mailto:oleksyk@oakland.edu)

^+^ these authors contributed equally to this work

**Table S1.** Variable sites, haplotypes and haplogroups in the 248bp fragment of the mitochondrial DNA D-loop of 162 Puerto Rican Paso Finos (PRPF) and 38 “Criollos” or the non-purebred (NPB) horses. Alleles identical to the reference haplotype (Hap_1*) are omitted. The haplotypes are cross-referenced to the reference haplogroup nomenclature used in Cieslak et al. (2010). The exact phylogenetic relationship between the groups can be seen in **Figure 1.**

| **mtDNA Haplotype** | **PRPF (%)** | **PR NPB (%)** | **Reference Haplogroup*** | **Locations of the Variable Sites** | | | | | | | | | | | | | | | | | | | | | | | |
| --- | --- | --- | --- | --- | --- | --- | --- | --- | --- | --- | --- | --- | --- | --- | --- | --- | --- | --- | --- | --- | --- | --- | --- | --- | --- | --- | --- |
|  |  |  |  | **15,494** | **15,495** | **15,496** | **15,521** | **15,526** | **15,533** | **15,534** | **15,536** | **15,540** | **15,542** | **15,595** | **15,600** | **15,601** | **15,602** | **15,603** | **15,617** | **15,635** | **15,649** | **15,659** | **15,666** | **15,703** | **15,718** | **15,720** | **15,740** |
| ***Hap_1**** | 8 | 5 | ***D2*** | T | T | A | G | T | A | C | T | A | C | A | G | T | C | T | T | C | A | T | G | T | C | G | A |
| ***Hap_2*** | 37 | 35 | ***X2*** | C | C | G | . | . | . | T | . | . | . | . | . | . | T | C | . | . | G | . | . | . | . | A | . |
| ***Hap_3*** | 2 | 4 | ***X2*** | C | C | G | . | . | . | T | . | . | . | . | . | . | . | C | . | . | G | . | . | . | . | A | . |
| ***Hap_4*** |  | 10 | ***B1*** | . | C | . | . | . | . | . | . | . | . | . | . | . | T | . | C | . | . | C | . | . | . | A | . |
| ***Hap_5*** | 16 | 3 | ***G3*** | . | C | . | A | . | . | . | . | . | . | . | . | . | T | . | . | . | . | . | . | . | . | A | . |
| ***Hap_6*** | 2 | 5 | ***X3*** | . | C | . | . | . | . | . | . | . | T | . | . | . | T | . | . | T | . | . | A | C | . | A | . |
| ***Hap_7*** | 13 | 22 | ***D3*** | . | C | . | . | . | . | . | . | . | . | . | . | . | . | . | . | . | . | . | A | . | . | A | . |
| ***Hap_8*** | 2 | 1 | ***K2*** | . | C | . | . | . | . | . | . | . | . | . | A | . | T | . | . | . | . | . | . | C | . | A | G |
| ***Hap_9*** | 8 | 4 | ***D2*** | . | C | . | . | . | . | . | . | . | . | . | . | . | . | . | . | . | . | . | . | . | . | . | . |
| ***Hap_10*** |  | 1 | ***X2*** | C | C | G | . | . | . | T | . | . | . | . | . | . | . | . | . | . | G | . | . | . | . | A | . |
| ***Hap_11*** | 3 | 4 | ***H1a*** | . | C | . | . | . | - | . | C | . | . | . | A | . | T | . | . | . | . | . | . | . | . | A | . |
| ***Hap_12*** |  | 1 | ***B1*** | . | C | . | . | . |  | . | . | . | . | G | . | . | T | . | C | . | . | C | . | . | . | A | . |
| ***Hap_13*** | 3 | 1 | ***X3*** | . | C | . | . | . |  | . | . | . | T | . | . | . | T | . | . | . | . | . | A | . | . | A | . |
| ***Hap_14*** |  | 1 | ***D3*** | . | C | . | A | . | . | . | . | . | . | . | . | . | . | . | . | . | . | . | A | . | . | A | . |
| ***Hap_15*** |  | 1 | ***H1a*** | . | . | . | . | . | C | . | C | . | . | . | A | . | T | . | . | . | . | . | . | . | . | A | . |
| ***Hap_16*** |  |  | ***X2*** | C | C | G | . | . | - | . | . | . | . | . | . | . | T | C | . | . | G | . | . | . | . | A | . |
| ***Hap_17*** | 3 | 1 | ***X4*** | . | C | . | . | C | . | . | . | G | . | . | . | . | T | . | . | . | G | . | . | . | T | A | . |
| ***Hap_18*** | 3 |  | ***H1*** | . | C | . | . | . | . | . | C | . | . | . | A | . | T | . | . | . | . | . | . | . | . | A | . |
| ***Hap_19*** |  | 1 | ***F*** | . | C | . | . | . | . | . | . | . | . | . | . | C | T | . | . | . | . | . | . | . | . | A | . |
| ***Hap_20*** |  | 1 | ***X1*** | . | . | . | . | . | . | T | . | . | . | . | . | . | T | C | . | . | G | . | . | . | . | A | . |

**Table S2.** Selected targets identified by genome wide **XP-EHH** and **RSB** tests (Figures 6 and 7). Genes in bold are described in Appendix 1. Negative values in the parentheses indicate direction of selection in plots (Figures 6 and 7).

| **REGION** | **GENE(S)** | **CHROMOSOME** | **POSITION** | **XP EHH** | | | **RSB** | | |
| --- | --- | --- | --- | --- | --- | --- | --- | --- | --- |
|  |  |  |  | **In PRPF*** | **In PR NRB** | **In PRPF vs. PR NRB** | **In PRPF*** | **In PR NRB** | **In PRPF vs. PR NRB** |
| *PRS1* | ***CHRM5*** | 1 | 154,082,628 | (5.12) |  | 5.75 | (4.51) |  | 5.50 |
| *PRS2* | *RNASE10, RNASE12* | 1 | 156,642,282 | (4.03) |  | 4.59 |  |  | - |
| *PRS3* | ***CYP2E1****, MIR8947* | 1 | 161,130,579 | (4.52) |  | 5.21 | (4.53) |  | 4.17 |
| *PRS4* | ***MYH7,*** *MIR208A, MIR208B* | 1 | 161,184,230 | (4.54) |  | 4.63 | (5.11) |  | 4.48 |
| *PRS5* | *MIR9047* | 1 | 161,817,069 |  |  | 4.59 |  |  | - |
| ***PRS6*** | ***STRN3, HEATR5A*** | 1 | 167,046,919 | (4.45) |  | 4.39 | (4.54) |  | 4.28 |
| *PRS7* | *MIR8990* | 7 | 40,423,783 |  |  | 4.58 |  |  | 4.35 |
| *PRS8* | ***HPGD****, MIR9108* | 11 | 25,516,779 |  |  | - |  |  | 4.23 |
| *PRS9* | ***SRSF1****, MIR9152* | 11 | 30,521,866 |  |  | 4.08 |  |  | - |
| *PRS10* | *PAM* | 14 | 66,106,336 |  |  | 4.37 |  |  | - |
| *PRS11* | *COMP, SERPINB1, MIR130B, MIR301B, MIR8923-1* | 15 | 61,299,263 |  |  | - | (4.11) |  | - |
| *PRS12* | *BLVRA, PDE4D, SYNE1* | 21 | 12,679,828 |  |  | - |  |  | 4.16 |
| *PRS13* | ***PRND, PRNP*** | 22 | 16,867,450 | (4.19) |  | 4.39 | (4.74) |  | 4.84 |
| *PRS14* | ***DLD, SNRPB2,*** *MIR1291B* | 22 | 31,942,581 |  |  | - | (4.37) |  | - |
| *PRS15* | *MIRLET7D, MIRLET7F* | 23 | 23,272,843 |  | (4.79) | - |  | (4.64) | - |
| *PRS16* | *TBCCD1, MIR10A, MIR8952* | 23 | 24,142,424 |  |  | - |  | (4.38) | - |

**Table S3.** Detailed description of the protein coding genes located in the putative selection regions in Puerto Rican Paso Fino genome

**Chromosome 1**

The muscarinic cholinergic receptor **CHRM5** belongs to a larger family of G protein-coupled receptors. The functional diversity of these receptors is defined by the binding of acetylcholine and includes cellular responses such as adenylate cyclase inhibition, phosphoinositide degeneration, and potassium channel mediation. Muscarinic receptors influence many effects of acetylcholine in the central and peripheral nervous system. The clinical implications of this receptor are unknown; however, stimulation of this receptor is known to increase cyclic AMP levels. [provided by RefSeq, Jul 2008]. **Diseases associated with CHRM5 include Schizophrenia.**

Cytochrome P450 Family 2 Subfamily E Member **CYP2E1** encodes a member of the cytochrome P450 superfamily of enzymes. The cytochrome P450 proteins are monooxygenases which catalyze many reactions involved in drug metabolism and synthesis of cholesterol, steroids and other lipids. This protein localizes to the endoplasmic reticulum and is induced by ethanol, the diabetic state, and starvation. The enzyme metabolizes both endogenous substrates, such as ethanol, acetone, and acetal, as well as exogenous substrates including benzene, carbon tetrachloride, ethylene glycol, and nitrosamines which are premutagens found in cigarette smoke. Due to its many substrates, this enzyme may be involved in such varied processes as gluconeogenesis, hepatic cirrhosis, diabetes, and cancer. [provided by RefSeq, Jul 2008]

Muscle myosin **MYH7** is a hexameric protein containing 2 heavy chain subunits, 2 alkali light chain subunits, and 2 regulatory light chain subunits. This gene encodes the beta (or slow) heavy chain subunit of cardiac myosin. It is expressed predominantly in normal human ventricle. It is also expressed in skeletal muscle tissues rich in slow-twitch type I muscle fibers. Changes in the relative abundance of this protein and the alpha (or fast) heavy subunit of cardiac myosin correlate with the contractile velocity of cardiac muscle. Its expression is also altered during thyroid hormone depletion and hemodynamic overloading. Mutations in this gene are associated with familial hypertrophic cardiomyopathy, myosin storage myopathy, dilated cardiomyopathy, and Laing early-onset distal myopathy. [provided by RefSeq, Jul 2008]

**HEATR5A (HEAT Repeat Containing 5A)** is a Protein Coding gene. Diseases associated with HEATR5A include Bardet-Biedl Syndrome 4 and Ceroid Lipofuscinosis, Neuronal, 6. Gene Ontology (GO) annotations related to this gene include binding. An important paralog of this gene is HEATR5B.

**STRN3 (Striatin 3)** is a Protein Coding gene. Diseases associated with STRN3 include Syndromic Intellectual Disability. Among its related pathways are Neurophysiological process Glutamate regulation of Dopamine D1A receptor signaling. Gene Ontology (GO) annotations related to this gene include DNA-binding transcription factor activity and calmodulin binding. An important paralog of this gene is STRN.

**Chromosome 11**

**SRSF1** gene encodes a member of the arginine/serine-rich splicing factor protein family. The encoded protein can either activate or repress splicing, depending on its phosphorylation state and its interaction partners. Multiple transcript variants have been found for this gene. There is a pseudogene of this gene on chromosome 13. [provided by RefSeq, Jun 2014]. **Diseases associated with SRSF1 include Retinitis Pigmentosa 4 and Homocystinuria.**

**Chromosome 14**

**PAM gene** encodes a multifunctional protein. The encoded preproprotein is proteolytically processed to generate the mature enzyme. This enzyme includes two domains with distinct catalytic activities, a peptidylglycine alpha-hydroxylating monooxygenase (PHM) domain and a peptidyl-alpha-hydroxyglycine alpha-amidating lyase (PAL) domain. These catalytic domains work sequentially to catalyze the conversion of neuroendocrine peptides to active alpha-amidated products. Alternative splicing results in multiple transcript variants, at least one of which encodes an isoform that is proteolytically processed. [provided by RefSeq, Jan 2016]. Diseases associated with PAM include Menkes Disease and Spinal Muscular Atrophy, Distal, X-Linked 3.

**Chromosome 22**

**DLD gene** encodes a member of the class-I pyridine nucleotide-disulfide oxidoreductase family. The encoded protein has been identified as a moonlighting protein based on its ability to perform mechanistically distinct functions. In homodimeric form, the encoded protein functions as a dehydrogenase and is found in several **multi-enzyme complexes that regulate energy metabolism. However, as a** monomer, this protein can function as a protease. Mutations in this gene have been identified in patients with E3-deficient maple syrup urine disease and lipoamide dehydrogenase deficiency. Alternative splicing results in multiple transcript variants. [provided by RefSeq, Jan 2014]. Mutant phenotypes in humans include **abnormalities in head and neck.**

**SRNPB2** gene encodes a protein associates with stem loop IV of U2 small nuclear ribonucleoprotein (U2 snRNP) in the presence of snRNP-A'. The encoded protein may play a role in pre-mRNA splicing. Autoantibodies from **patients with systemic lupus erythematosus** frequently recognize epitopes on the encoded protein. Two transcript variants encoding the same protein have been found for this gene. [provided by RefSeq, Jul 2008]

**PRNP gene** codes for a membrane glycosylphosphatidylinositol-anchored glycoprotein that tends to aggregate into rod-like structures. The encoded protein contains a highly unstable region of five tandem octapeptide repeats. In humans this gene is found on chromosome 20, approximately 20 kbp upstream of a gene which encodes a biochemically and structurally similar protein to the one encoded by this gene.

Mutations in the repeat region as well as elsewhere in this gene have **been associated with Creutzfeldt-Jakob disease, fatal familial insomnia, Gerstmann-Straussler disease, Huntington disease-like 1, and kuru.** An overlapping open reading frame has been found for this gene that encodes a smaller, structurally unrelated protein, AltPrp. Alternative splicing results in multiple transcript variants.

May play a role in neuronal development and synaptic plasticity. May be required for neuronal myelin sheath maintenance. May promote myelin homeostasis through acting as an agonist for ADGRG6 receptor. May play a role in iron uptake and iron homeostasis

**Table S4.** The values of pairwise IBD proportions between the three purebred Paso Fino (PRPF) horses collected in this study (OS_PASO) individually compared to all the other Paso Fino horses used in this study (OS PASO and other PRPF from (Petersen et al., 2013)). Pairwise IBD values higher than 0.125, corresponding to the relationships between the third-degree relatives or higher are highlighted in yellow. No first degree relatives were detected, but two of the three PRPF horses from our study (OS_PASO) are related at the IBD>0.271, corresponding to the relationships between the second-degree relatives. The IBD values were calculated in PLINK (Chang et al., 2015) according to the equations originally developed by S. Wright (Malécot, 1948; Wright, 1922).

| **PRPF ID** | **PURCID3** | **PURCMY11** | **PURPNC2** |
| --- | --- | --- | --- |
| **OS_PASO** |  |  |  |
| PURCID3 | **-** | **0.271** | 0 |
| PURCMY11 | **-** | - | 0.111 |
| **PRPF** |  |  |  |
| RP006 | 0 | 0 | **0.127** |
| RP007 | 0 | 0.099 | 0.097 |
| RP008 | 0 | 0.108 | **0.153** |
| RP010 | 0 | 0.068 | 0.051 |
| RP014 | **0.219** | **0.210** | 0.088 |
| RP458 | **0.203** | **0.177** | 0.109 |
| RP459 | 0 | 0 | 0.079 |
| RP461 | 0 | 0.123 | 0.095 |
| RP462 | 0 | **0.172** | 0.099 |
| RP497 | 0 | 0 | 0 |
| RP499 | **0.166** | **0.182** | 0.117 |
| RP500 | **0.186** | **0.192** | 0.102 |
| RP502 | 0 | 0.109 | 0.119 |
| RP503 | 0 | 0 | 0 |
| RP504 | **0.221** | **0.201** | 0.121 |
| RP505 | 0.000 | **0.137** | 0.094 |
| RP506 | 0 | **0.133** | 0.085 |
| RP507 | 0 | 0 | 0.069 |
| RP508 | 0 | 0.000 | 0 |
| RP889 | 0 | **0.145** | 0.093 |

**Table S5.** The values of pairwise IBD proportions between purebred Paso Fino (PRPF) Paso Fino horses from Petersen et al. (2013). The average IBD was 0.145±0.004 indicating moderate amount of inbreeding similar to other breeds (ex. Peruvian Paso in the same study, 0.14±0.002). No first degree relatives were detected. Pairwise IBD values higher than 0.125, corresponding to the relationships between the third-degree relatives or higher are highlighted in yellow. Pairwise IBD values equal or higher than 0.25, corresponding to the relationships between the second-degree relatives or higher, are highlighted in pink. The IBD values were calculated in PLINK (Chang et al., 2015)according to the equations originally developed by S. Wright (Malécot, 1948; Wright, 1922).

| **PRPF**  **ID** | **RP007** | **RP008** | **RP010** | **RP014** | **RP458** | **RP459** | **RP461** | **RP462** | **RP497** | **RP499** | **RP500** | **RP502** | **RP503** | **RP504** | **RP505** | **RP506** | **RP507** | **RP508** | **RP889** |
| --- | --- | --- | --- | --- | --- | --- | --- | --- | --- | --- | --- | --- | --- | --- | --- | --- | --- | --- | --- |
| **RP006** | **0.162** | **0.136** | 0.071 | 0.116 | **0.137** | 0.123 | **0.136** | **0.129** | **0.145** | **0.161** | **0.156** | **0.153** | **0.130** | **0.138** | 0.118 | **0.149** | 0.075 | 0 | 0.112 |
| **RP007** |  | **0.174** | 0.111 | **0.175** | **0.232** | 0.123 | **0.263** | **0.160** | **0.254** | **0.234** | **0.191** | **0.167** | **0.162** | **0.202** | **0.186** | **0.193** | 0.118 | **0.179** | **0.139** |
| **RP008** |  |  | **0.146** | **0.199** | **0.227** | 0.104 | **0.143** | **0.165** | **0.157** | **0.175** | **0.188** | **0.208** | **0.221** | **0.236** | **0.213** | **0.181** | 0.097 | **0.184** | **0.148** |
| **RP010** |  |  |  | **0.133** | **0.132** | 0.068 | 0.105 | **0.129** | 0.104 | **0.130** | 0.107 | 0.119 | 0.089 | 0.082 | 0.104 | 0.106 | 0.068 | 0.087 | 0.093 |
| **RP014** |  |  |  |  | **0.371** | 0.091 | **0.162** | **0.280** | **0.288** | **0.294** | **0.313** | **0.210** | **0.181** | **0.349** | **0.165** | **0.240** | 0.086 | **0.150** | 0.116 |
| **RP458** |  |  |  |  |  | 0.103 | **0.159** | **0.228** | **0.219** | **0.267** | **0.269** | **0.253** | **0.180** | **0.315** | **0.232** | **0.235** | 0.113 | **0.162** | 0.117 |
| **RP459** |  |  |  |  |  |  | 0.086 | 0.123 | 0.107 | 0.122 | 0.105 | **0.137** | 0.107 | **0.141** | 0.115 | 0.122 | 0.091 | 0.000 | 0.140 |
| **RP461** |  |  |  |  |  |  |  | 0.122 | **0.229** | **0.159** | **0.163** | 0.119 | **0.143** | **0.170** | **0.192** | **0.145** | 0.116 | **0.172** | 0.099 |
| **RP462** |  |  |  |  |  |  |  |  | **0.145** | **0.279** | **0.215** | **0.165** | **0.156** | **0.258** | **0.206** | **0.213** | 0.116 | **0.140** | **0.197** |
| **RP497** |  |  |  |  |  |  |  |  |  | **0.170** | **0.225** | **0.204** | **0.180** | **0.178** | **0.184** | **0.186** | 0.070 | **0.175** | **0.155** |
| **RP499** |  |  |  |  |  |  |  |  |  |  | **0.258** | **0.187** | **0.241** | **0.355** | **0.179** | **0.168** | 0.097 | **0.195** | **0.178** |
| **RP500** |  |  |  |  |  |  |  |  |  |  |  | **0.223** | **0.150** | **0.350** | **0.211** | **0.226** | 0.079 | **0.181** | **0.145** |
| **RP502** |  |  |  |  |  |  |  |  |  |  |  |  | **0.168** | **0.232** | **0.202** | **0.216** | 0.098 | **0.195** | **0.139** |
| **RP503** |  |  |  |  |  |  |  |  |  |  |  |  |  | **0.243** | **0.206** | **0.209** | 0.092 | **0.232** | **0.147** |
| **RP504** |  |  |  |  |  |  |  |  |  |  |  |  |  |  | **0.254** | **0.230** | 0.110 | **0.201** | **0.149** |
| **RP505** |  |  |  |  |  |  |  |  |  |  |  |  |  |  |  | **0.200** | 0.088 | **0.292** | **0.177** |
| **RP506** |  |  |  |  |  |  |  |  |  |  |  |  |  |  |  |  | 0.112 | **0.148** | **0.151** |
| **RP507** |  |  |  |  |  |  |  |  |  |  |  |  |  |  |  |  |  | 0 | 0.079 |
| **RP508** |  |  |  |  |  |  |  |  |  |  |  |  |  |  |  |  |  |  | **0.175** |

**Table S6.** The values of pairwise IBD proportions between nonpurebred horses from Puerto Rico (PR NPB). The average IBD was 0.02±0.002, indicating low amounts of inbreeding. Pairwise IBD values higher than 0.125, corresponding to the relationships between the third-degree relatives or higher are highlighted in yellow. Pairwise IBD values equal or higher than 0.25, corresponding to the relationships between the second-degree relatives or higher, are highlighted in pink. The IBD values were calculated in PLINK (Chang et al., 2015)according to the equations originally developed by S. Wright (Malécot, 1948; Wright, 1922).

| **NRB “Criollo” ID** | **AGD5** | **AGD6** | **CID10** | **CIDQUILAN** | **CMYTORO1** | **CRL6** | **FJD2** | **GYB1** | **LJS4** | **LPS1** | **LQL16** | **LQL6** | **LRS8** | **PNC2** | **PNC4** | **PNC5** | **QBS2** | **RGD1** | **RNC6** | **UTD1** |
| --- | --- | --- | --- | --- | --- | --- | --- | --- | --- | --- | --- | --- | --- | --- | --- | --- | --- | --- | --- | --- |
| **AG8** | 0.03 | 0.04 | 0.05 | 0.05 | 0 | 0.03 | 0 | 0.04 | 0.02 | 0.03 | 0 | 0.02 | 0.03 | 0.03 | 0.03 | 0.02 | 0.04 | 0 | 0.03 | 0.05 |
| **AGD5** |  | 0.04 | 0 | 0.05 | 0 | 0.04 | 0.03 | 0.05 | 0.02 | 0.04 | 0 | 0.08 | 0.04 | 0.06 | 0.03 | 0.04 | 0.05 | 0 | 0.02 | 0.05 |
| **AGD6** |  |  | 0.04 | 0.04 | 0 | 0.03 | 0.04 | 0 | 0 | 0.03 | 0.04 | 0.04 | 0.04 | 0.03 | 0.04 | 0.04 | 0.03 | 0 | 0.03 | 0.04 |
| **CID10** |  |  |  | 0 | 0 | 0.06 | 0 | 0 | 0 | 0.05 | 0 | 0.05 | 0 | 0 | 0 | 0 | 0.03 | 0 | 0 | 0 |
| **CIDQUILAN** |  |  |  |  | 0 | 0.05 | 0.07 | 0 | 0.04 | 0.06 | 0 | 0.09 | 0.06 | 0.07 | 0.06 | 0.04 | 0.04 | 0 | 0.03 | 0.07 |
| **CMYTORO1** |  |  |  |  |  | 0 | 0.00 | 0 | 0 | 0 | 0 | 0 | 0 | 0 | 0.00 | 0 | 0 | 0 | 0 | 0 |
| **CRL6** |  |  |  |  |  |  | 0.05 | 0 | 0 | 0.04 | 0 | 0.04 | 0.04 | 0.05 | 0 | 0.03 | 0.05 | 0 | 0 | 0.04 |
| **FJD2** |  |  |  |  |  |  |  | 0 | 0 | 0.05 | 0 | 0.00 | 0 | 0.05 | 0 | 0 | 0 | 0 | 0 | 0.06 |
| **GYB1** |  |  |  |  |  |  |  |  | 0 | 0 | 0 | 0.04 | 0.05 | 0 | 0 | 0 | 0 | 0 | 0 | 0 |
| **LJS4** |  |  |  |  |  |  |  |  |  | 0 | 0 | 0.02 | 0 | 0 | 0.04 | 0 | 0.02 | 0 | 0 | 0 |
| **LPS1** |  |  |  |  |  |  |  |  |  |  | 0.03 | 0.04 | 0.04 | 0.05 | 0.07 | 0 | 0.04 | 0 | 0.03 | 0.04 |
| **LQL16** |  |  |  |  |  |  |  |  |  |  |  | 0 | 0 | 0 | 0 | 0.02 | 0.03 | 0 | 0 | 0 |
| **LQL6** |  |  |  |  |  |  |  |  |  |  |  |  | 0.05 | 0.04 | 0.04 | 0.04 | 0.04 | 0.08 | 0.04 | 0.04 |
| **LRS8** |  |  |  |  |  |  |  |  |  |  |  |  |  | 0.04 | 0.05 | 0.02 | 0.04 | 0 | 0.04 | 0.05 |
| **PNC2** |  |  |  |  |  |  |  |  |  |  |  |  |  |  | 0.05 | 0 | 0.05 | 0 | 0 | 0.06 |
| **PNC4** |  |  |  |  |  |  |  |  |  |  |  |  |  |  |  | 0.04 | 0.04 | 0 | 0 | 0 |
| **PNC5** |  |  |  |  |  |  |  |  |  |  |  |  |  |  |  |  | 0.02 | 0 | 0.04 | 0 |
| **QBS2** |  |  |  |  |  |  |  |  |  |  |  |  |  |  |  |  |  | 0 | 0.01 | 0.03 |
| **RGD1** |  |  |  |  |  |  |  |  |  |  |  |  |  |  |  |  |  |  | 0 | 0 |
| **RNC6** |  |  |  |  |  |  |  |  |  |  |  |  |  |  |  |  |  |  |  | 0 |


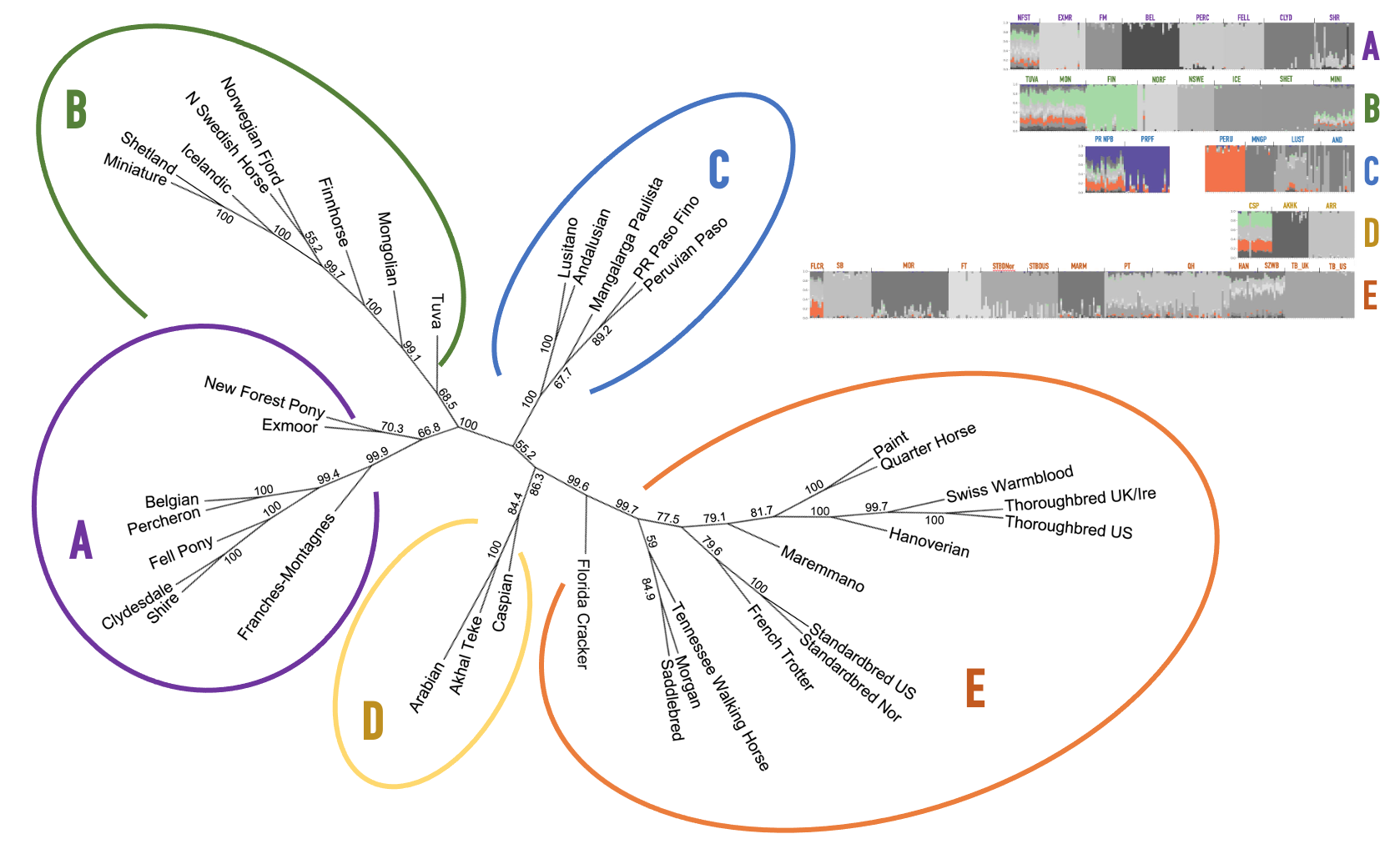


**Figure S1.** The neighbor joining tree calculated from SNP frequencies in 38 horse populations modified from Petersen at al. (2013) and the designation of five clusters used in this analysis (**Figures 2 and 3**). The insert shows correspondence of clades A, B, C, D and E to the groupings in **Figure 2** showing Principal Component Analysis (PCA) plot of horse breeds, and **Figure 3** showing structure of the horse populations used in this study.

**Figure S2**. Admixture plot of horse breeds masking components that are rare in the Puerto Rican Non Pure Breed (NPB) *Criollo* horses (<5 % of population structure, in gray). As a result, the population components that may have been shared are clearly visible. Data on Puerto Rican Paso Fino and Criollo horses are from Illumina Neogen Equine Community Array genotyped in this study, other genotypes are from Petersen at al. (2013).

**Figure S3**. Admixture Graph of horse breeds Data on PRPF and Criollo horses are from Illumina Neogen Equine Community Array genotyped in study, other genotypes are from Petersen at al. (2013). The letter codes (in capitals) indicate groups highlighted in **Figures 2 and S1**.


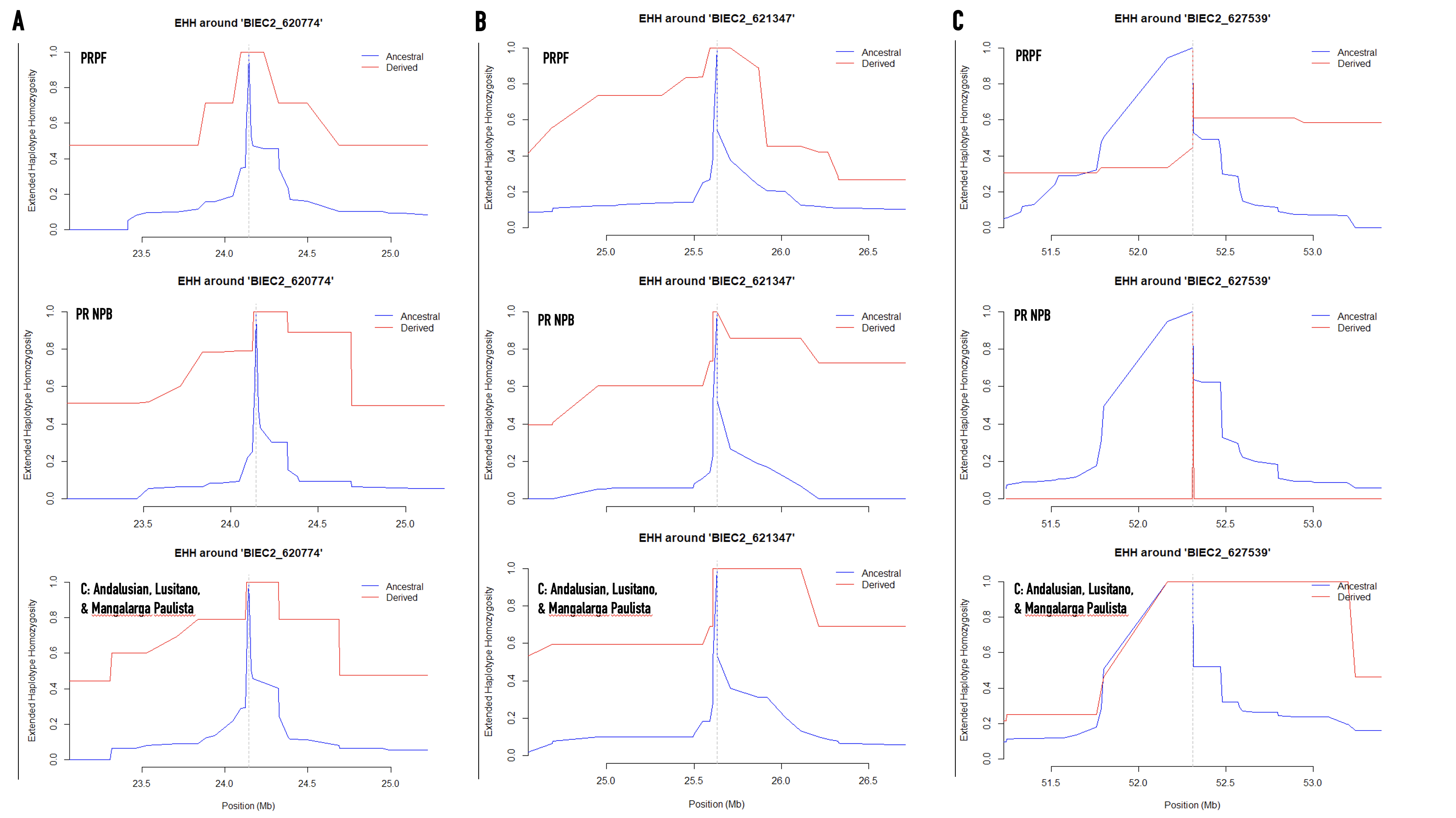


**Figure S4.** Extended Haplotype Homozygosity (EHH) decay graph for the regions around the DMRT3_Ser301STOP mutation on Chromosome 23 associated with the Paso Gait (loci BIEC2 627539, BIEC2 621347, BIEC2 620774) for A. PRPF, B). PR NRB and C) breeds in the C clade (Figure S2) containing Andalusian, Lusitano, and Mangalarga Paulista. The extended homozygosity is more pronounced for the haplotypes containing the nonsense (A) allele (derived, red line) in the two horse breeds from Puerto Rico.

**
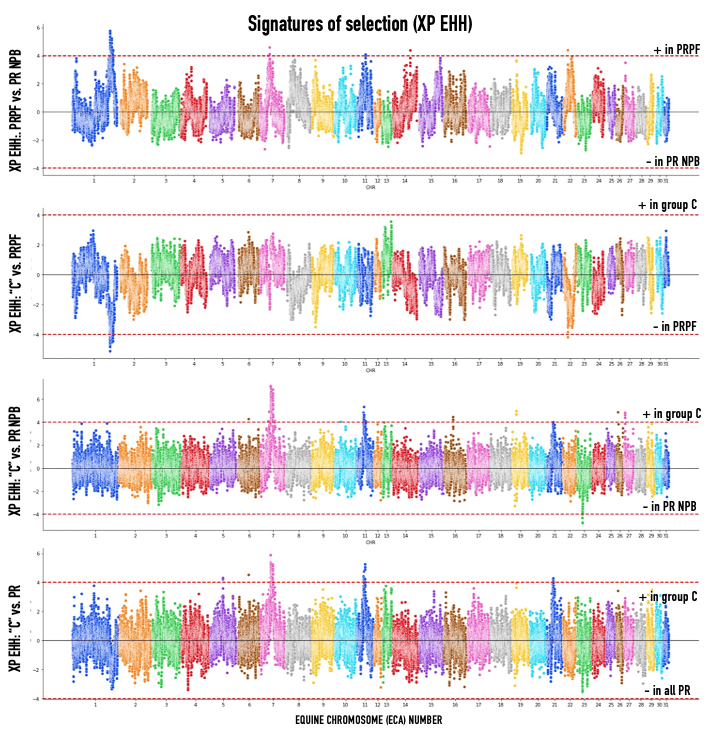
**

**Figure S5.** Signatures of selection in Puerto Rico Paso Fino (PRPF) and PR nonpurebred (NPB) horse genomes based on XP EHH scans of the combined samples in this study. Selection tests comparing PRPF and PR NPB horse genomes to the rest of the breeds in the C clade (Lusitano, Andalusian and Mangalanga Paulista; Figure S2). Genes with XP EHH values > 4 are displayed on the graph in Figure 6. The locations of the selection regions are listed in Table S1.

**
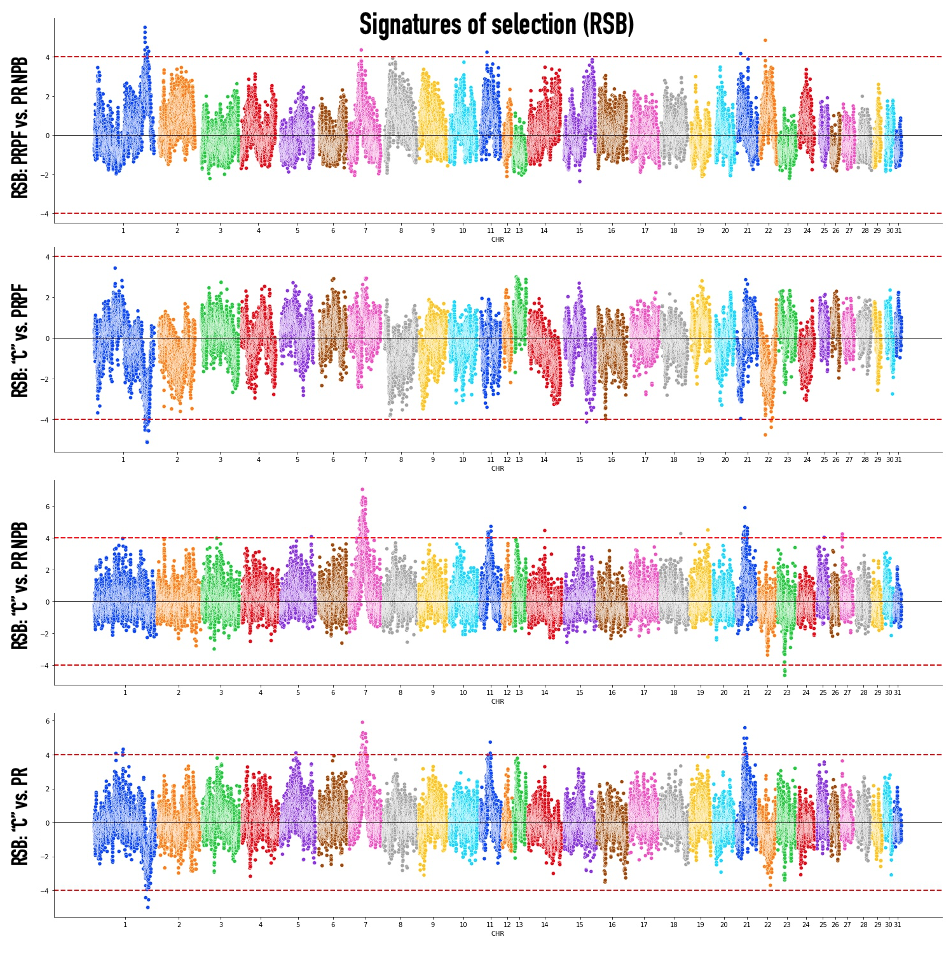
**

**Figure S6.** Signatures of selection in Puerto Rico Paso Fino (PRPF) and PR nonpurebred (NPB) horse genomes based on RSB scans of the combined samples in this study. Selection tests comparing PRPF and PR NPB horse genomes to the rest of the breeds in the C clade (Lusitano, Andalusian and Mangalanga Paulista; Figure S2). The locations of the selection regions are listed in Table S1. Genes with RSB values > 4 are displayed on the graph in Figure 6. The locations of the selection regions are listed in Table S1.


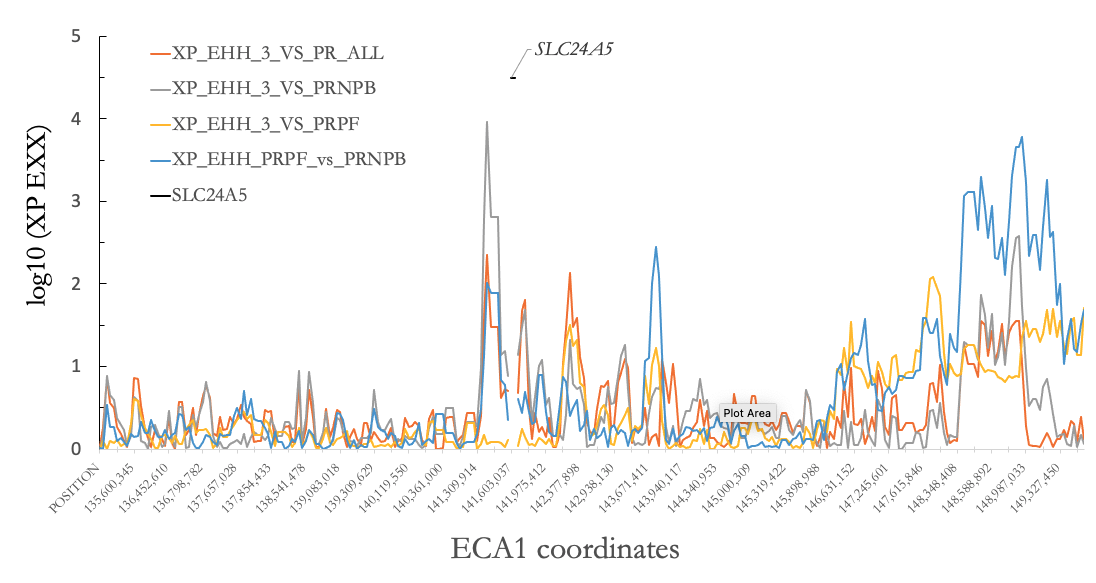


**Figure S7.** Signatures of selection (XP EHH) in the chromosomal neighborhood around the *SLC24A5* gene in our PRPF lineages.


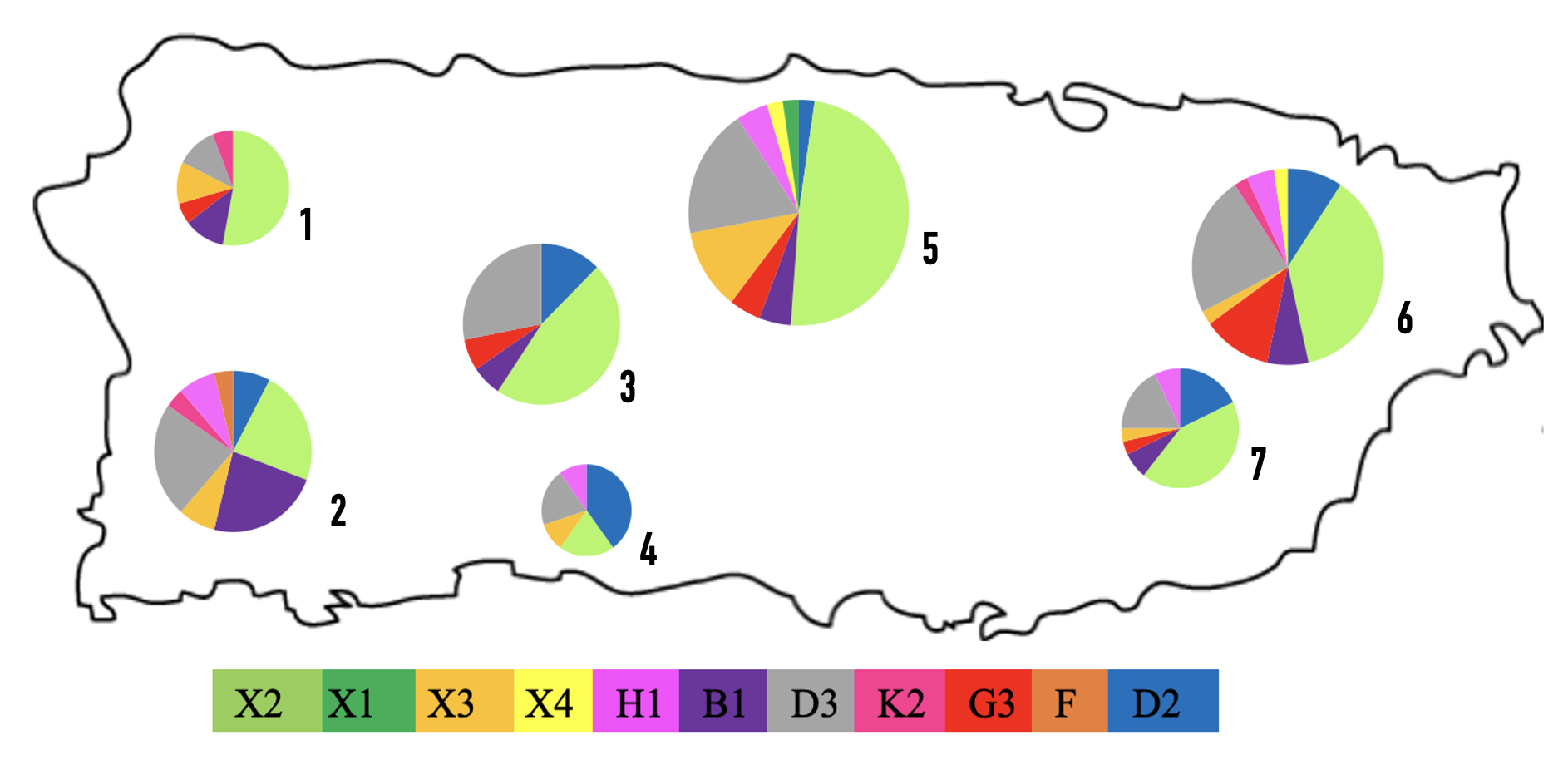


**Figure S8**. Sampling of the 200 horses across Puerto Rico, and frequencies of mitochondrial haplotypes within 7groups of sampling sites: 1. Aguada, Aguadilla, Rincón; 2. Mayagüez, Hormigueros, Lajas; 3. Camuy Hatillo, Lares, Utuado; 4. Ponce; 5. Barceloneta, ManatíToa Baja, Guaynabo; 6. Carolina, Trujillo Alto, Loíza, Canovanas, , Río Grande, Luquillo, Fajardo; 7. Humacao, Las Piedras. The size of the pie chart is proportional to the number of samples collected. The haplotypes are grouped according to the known haplogroups (Cieslak et al., 2010). Full results of the haplotype analysis are presented in the Table S1.
